# A ZEB1/p53 signaling axis in stromal fibroblasts promotes mammary epithelial tumours

**DOI:** 10.1038/s41467-019-11278-7

**Published:** 2019-07-19

**Authors:** Rong Fu, Chen-Feng Han, Ting Ni, Lei Di, Li-Juan Liu, Wen-Cong Lv, Yan-Ran Bi, Nan Jiang, Yin He, Hong-Mei Li, Shui Wang, Hui Xie, Bao-An Chen, Xiao-Sheng Wang, Stephen J. Weiss, Tao Lu, Qing-Long Guo, Zhao-Qiu Wu

**Affiliations:** 1 State Key Laboratory of Natural Medicines, Jiangsu Key Laboratory of Carcinogenesis and Intervention, School of Basic Medicine and Clinical Pharmacy, China Pharmaceutical University, Nanjing, 211198 China; 2 State Key Laboratory of Natural Medicines, Laboratory of Molecular Design and Drug Discovery, School of Science, China Pharmaceutical University, Nanjing, 211198 China; 3 Division of Breast Oncology, The First Affiliated Hospital, Nanjing Medical University, Nanjing, 210036 China; 4 Division of Hematology and Oncology, The Affiliated Zhong-Da Hospital, Southeast University, Nanjing, 210009 China; 5 The Life Sciences Institute, Rogel Cancer Center, Department of Internal Medicine, University of Michigan, Ann Arbor, MI 48109 USA

**Keywords:** Breast cancer, Cancer microenvironment

## Abstract

Accumulating evidence indicates that the zinc-finger transcription factor ZEB1 is predominantly expressed in the stroma of several tumours. However, the role of stromal ZEB1 in tumour progression remains unexplored. In this study, while interrogating human databases, we uncover a remarkable decrease in relapse-free survival of breast cancer patients expressing high *ZEB1* levels in the stroma. Using a mouse model of breast cancer, we show that *ZEB1* inactivation in stromal fibroblasts suppresses tumour initiation, progression and metastasis. We associate this with reduced extracellular matrix remodeling, immune cell infiltration and decreased angiogenesis. *ZEB1* deletion in stromal fibroblasts increases acetylation, expression and recruitment of p53 to *FGF2/7*, *VEGF* and *IL6* promoters, thereby reducing their production and secretion into the surrounding stroma. Importantly, *p53* ablation in *ZEB1* stroma-deleted mammary tumours sufficiently recovers the impaired cancer growth and progression. Our findings identify the ZEB1/p53 axis as a stroma-specific signaling pathway that promotes mammary epithelial tumours.

## Abstract (web summary)

In epithelial cells Zeb1 is involved in the epithelial to mesenchymal transition. In this study, the authors show in a mouse model of breast cancer, that Zeb1 expression in stromal cells is required for tumour formation and metastasis.

## Introduction

Genetic or epigenetic alterations in the tumour epithelial cells remain an important driver of cancer^1,2^. However, the surrounding tumour stroma, a complex microenvironment that the tumour epithelial cells live in, may also coevolve into an activated state during the tumourigenic process^3–7^. The reactive stroma functions as a key driver of tumour progression from initiation to metastasis, although relatively little is known about the signalling pathways that mediate the continuous crosstalk between the stromal and epithelial compartments^3–7^. The stromal cancer-associated fibroblast (CAF) is the most prominent cell type within the reactive tumour stroma, and is proposed to be a key mediator of the stromal–epithelial crosstalk^7–12^. Stromal CAFs are known to support many different aspects of tumour epithelial growth, progression and metastasis through secretion of a variety of signalling molecules, including growth-stimulatory, pro-angiogenic, immune-modulatory and pro-invasive factors as well as extracellular matrix (ECM) components^7–12^.

The zinc-finger transcription factor ZEB1 has been widely recognised as an important driver of tumour invasion, distant metastasis, drug resistance and radioresistance by inducing the epithelial-to-mesenchymal transition programme (EMT) in tumour epithelial cells^13–18^. However, accumulating evidence indicates that ZEB1 protein is predominantly present in tumour stromal cells in various human cancer samples, including breast cancer^19–22^. The role for tumour stroma-derived ZEB1 in mammary tumour growth, progression and metastasis remains unexplored. In this study, we have examined the expressing pattern of ZEB1 in different subtypes of human and mouse breast cancers. In fact, we find that *ZEB1* ablation in stromal CAFs increases acetylation, expression and recruitment of p53 to *FGF2*, *FGF7*, *VEGF* and *IL6* promoters and thus reduces their productions and secretions to the surrounding stroma, thereby creating a tumour-suppressive microenvironment that inhibits breast cancer growth and progression. The concomitant inactivation of stromal fibroblast-derived *p53* in *ZEB1* stroma-deleted mammary tumours efficiently recovers the impaired cancer growth and progression. In summary, we conclude that the stromal ZEB1/p53 signalling axis promotes mammary epithelial tumours in a paracrine fashion. Our findings suggest that genetic or pharmacological inhibition of tumour stromal ZEB1 or ZEB1/p53 interactions could be beneficial in combination with conventional tumour epithelial-targeted therapies.

## Results

### Stromal ZEB1 levels are increased in breast tumours

**Fig. 1:**
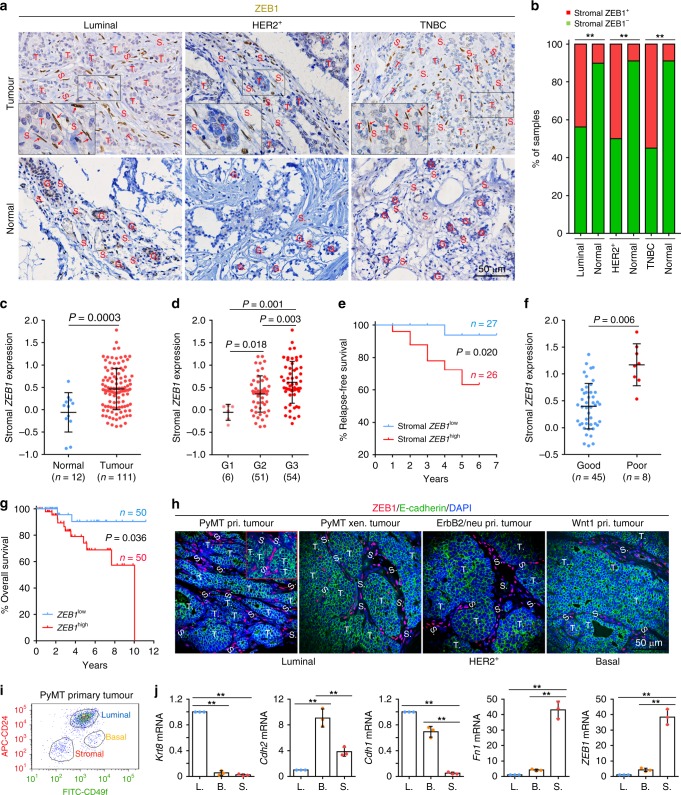
Stromal ZEB1 levels are increased in breast tumours and correlate negatively with patient survival. **a** Representative examples of ZEB1 staining of the human breast tissue arrays consisting of 98 luminal, 22 HER2^+^ and 47 TNBC tumour samples, as well as the corresponding adjacent normal samples. S stroma, T tumour. Insets display higher magnifications of boxed areas. Red arrows depict ZEB1^+^ tumour stromal cells. **b** Quantification of ZEB1 staining in the stroma of normal and breast cancer samples as described in **a**. Fisher’s exact test. **c**, **d** Bioinformatic analysis of *ZEB1* mRNA levels in the stroma of normal and breast cancer samples. The data were collected from a public data set of stromal gene expression in human breast cancer (GSE9014). Two-sided Student’s *t* test (**c**) and two-way ANOVA test (**d**). **e** Kaplan–Meier survival analysis of the relationship between relapse-free survival rate and stromal *ZEB1* expression level in breast cancer patients. Log-rank test. **f** Dot plots for stromal *ZEB1* mRNA levels across the clinical outcome clusters as described in **e**. Two-sided Student’s *t* test. **g** Kaplan–Meier survival analysis of the relationship between overall survival rate and *ZEB1* expression level in breast cancer patients. Patient samples with the highest stromal abundances were collected from TCGA and METABRIC data sets. Log-rank test. **h** Immunofluorescent staining of ZEB1 and E-cadherin in murine mammary tumours induced by *PyMT*, *ErbB2/neu* and *Wnt1* oncogenes (images are representative of images from five mice). Inset displays staining of lung metastasised tumours from MMTV-PyMT mice. Nuclei are counterstained with DAPI. Pri primary, Xen xenografted, S stroma, T tumour. **i** FACS-sorted cell lineages of MMTV-PyMT primary tumours. Luminal, basal and stromal cells are CD31^-^CD45^−^Ter119^−^(LIN^−^)DAPI^−^CD49f^low^CD24^+^, LIN^−^DAPI^−^CD49f^+^CD24^low^ and LIN^−^DAPI^−^CD49f^−^CD24^−^, respectively. The plot shown is representative of plots from three independent experiments. **j** RT-qPCR analyses of the expressions of *ZEB1* and specific markers for each cell population (*n* = 3 independent experiments): *keratin 8* (*Krt8*; L, luminal), *N-cadherin* (*Cdh2*; B, basal), *E-cadherin* (*Cdh1*; L + B) and *fibronectin* (*Fn1*; S, stromal). Two-way ANOVA test. All data are represented as mean ± s.d. **P < 0.01. The source data are provided as a Source Data file

To determine the expression pattern of ZEB1 in different subtypes of human breast cancer, we performed immunostaining of ZEB1 in the tissue arrays consisting of 98 luminal (ER and/or PR positive, HER2 negative or positive), 22 HER2^+^ (ER and PR negative, HER2 positive) and 47 triple-negative breast cancer (TNBC; ER and PR negative, HER2 negative) tumour samples, as well as the matched normal samples. We found that ZEB1 protein was primarily present in the stromal compartment, but was largely absent in the epithelial compartment of luminal, HER2^+^ and TNBC tumours (Fig. 1a). Stromal ZEB1 was present in 43.8% (43/98) of luminal, 50.0% (11/22) of HER2^+^ as well as 55.3% (26/47) of TNBC tumours, whereas it was detected in 10% or less of matched normal breast tissues (Fig. 1b). Bioinformatic analysis of a public human breast cancer data set (GSE9014) of stromal gene expression revealed that *ZEB1* expression levels in the tumour stroma were significantly higher than in the normal stroma, and were markedly increased upon tumour progression (Fig. 1c, d). Moreover, we identified a significantly reverse relationship between stromal *ZEB1* levels and relapse-free survival of patients and found that stromal *ZEB1* levels were markedly elevated in poor-outcome patients (Fig. 1e, f). While interrogating the Cancer Genome Atlas (TCGA) and the Molecular Taxonomy of Breast Cancer International Consortium (METABRIC) data sets, we uncovered a significant association between *ZEB1* levels and the tumour stromal abundances (Supplementary Fig. 1a, b). We further analysed the patient samples with the highest stromal abundances in the data sets and found that *ZEB1* levels were negatively correlated with overall survival of patients (Fig. 1g). To further determine the expression pattern of ZEB1 in mouse breast cancer, we performed immunostaining of mammary tumours from MMTV-PyMT, MMTV-ErbB2/neu and MMTV-Wnt1 transgenic mice, which spontaneously develop luminal B, HER2^+^ and basal subtype of breast cancer, respectively^23–25^. We found that ZEB1 was uniformly and predominantly expressed in the stromal compartment of primary, xenografted and metastasised mammary tumours (Fig. 1h), a finding consistent with ZEB1 expression in human breast cancer (Fig. 1a). Furthermore, fluorescence-activated cell sorting (FACS) analysis^26^ of PyMT-induced mammary tumours showed that *ZEB1* expression was highly enriched in the stromal fibroblasts (i.e., lineage-negative stromal CAFs) compared with luminal or basal epithelial cells (Fig. 1i, j). Reverse transcription quantitative PCR (RT-qPCR) analysis revealed that transcripts for the luminal marker keratin 8, the basal marker N-cadherin, the luminal/basal marker E-cadherin and the stromal marker fibronectin were enriched in their corresponding cell subpopulations, demonstrating successful cell fractionation (Fig. 1j). Collectively, these data suggest that ZEB1 is predominantly expressed in breast tumour stroma, and increased expression of stromal *ZEB1* worsens overall survival and relapse-free survival rates in breast cancer patients.

### Deletion of *ZEB1* inhibits mammary epithelial tumour formation

**Fig. 2:**
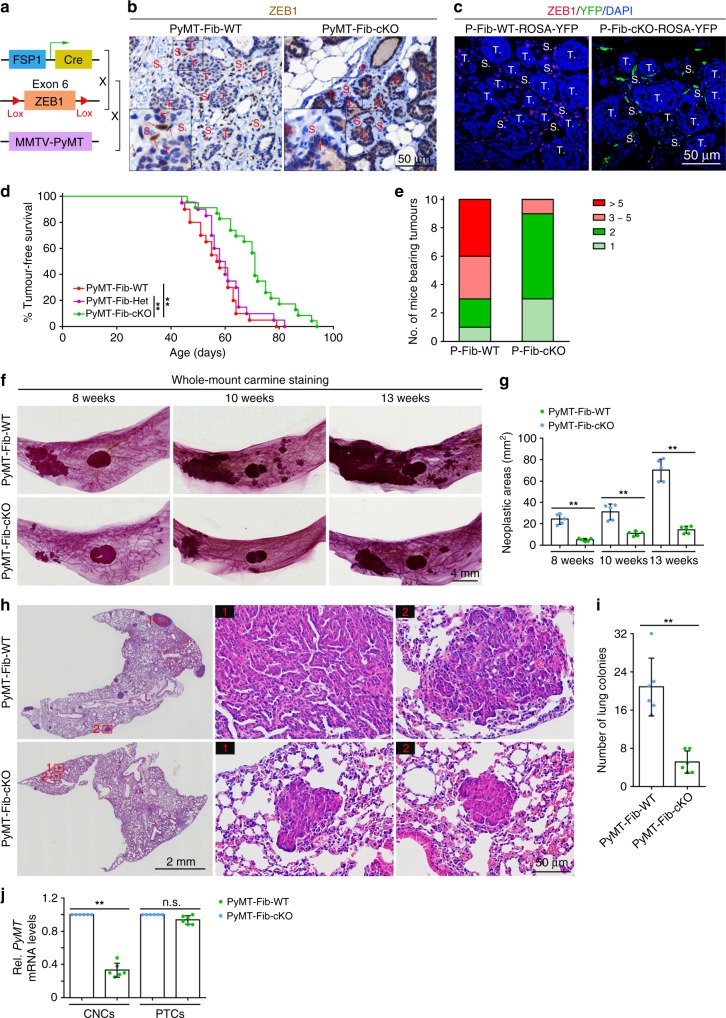
*ZEB1* deletion in stromal fibroblasts suppresses *PyMT* tumour formation, growth and metastasis. **a** The scheme of the transgenic mouse model for breast cancer. **b** Immunohistochemical staining of ZEB1 in PyMT-Fib-WT and -cKO tumours (images are representative of images from five mice). Insets display higher magnifications of boxed areas. **c** Immunofluorescence staining of ZEB1 and GFP in primary tumours of PyMT-Fib-WT-ROSA-YFP and PyMT-Fib-cKO-ROSA-YFP reporter mice (images are representative of images from five mice). Nuclei are counterstained with DAPI. **d** Kaplan–Meier analysis of mammary tumour progression in PyMT-Fib-WT (20 mice), -Het (20 mice) and -cKO (23 mice) females. Log-rank test. **e** The number of PyMT-Fib-WT and -cKO mice at 16 weeks of age (10 mice, each) that developed indicated numbers of palpable tumours. **f** Whole-mount Carmine red staining of neoplastic mammary glands from PyMT-Fib-WT and -cKO mice at 8, 10 and 13 weeks of age (images are representative of images from five mice). **g** Quantification of neoplastic areas in the mammary glands as shown in **f** (*n* = 5 independent experiments). Two-sided Student’s *t* test. **h** Haematoxylin and eosin (H&E) staining of lungs from PyMT-Fib-WT and -cKO mice at 9 weeks post detection of mammary tumours (six mice, each). Magnified areas of boxed sections are shown in right panels. **i** The number of lung colonies in the cohorts as shown in **h**. Two-sided Student’s *t* test. **j** RT-qPCR analyses of *PyMT* expression levels in circulating nucleated cells (CNCs) and primary tumour cells (PTCs) prepared from PyMT-Fib-WT and -cKO mice (*n* = 6 independent experiments). Two-sided Student’s *t* test. All data are represented as mean ± s.d. **P < 0.01, n.s. not significant. The source data are provided as a Source Data file

We first determined whether stromal fibroblast-derived ZEB1 plays a role in normal mammary gland development. We generated ZEB1^flox/flox^ mice (Supplementary Fig. 2a–e) and crossed this transgenic line with FSP1 (fibroblast-specific protein-1)-Cre mice^27,28^ to create female cohorts with genotypes of ZEB1^fl/fl^ (designated Fib-WT mice) and FSP1-Cre;ZEB1^fl/fl^ (Fib-cKO mice). In Fib-cKO-ROSA-YFP report mice, YFP expression was detected in the stromal compartment of mammary glands, complemented by the absence of ZEB1 protein in YFP^+^ stromal cells (Supplementary Fig. 2f). Proliferative cells were exclusively found in the epithelial compartment of mammary glands, and numbers of proliferative cells were comparable in Fib-WT and Fib-cKO mammary glands (Supplementary Fig. 2g). Consistently, we did not observe any phenotypic difference between Fib-WT and Fib-cKO mammary glands examined, during either puberty or pregnancy (Supplementary Fig. 2h). These results suggest that stromal fibroblast-derived ZEB1 is not required for mammary gland development. To further examine the impact of stromal fibroblast-derived ZEB1 on mammary tumour progression, we crossed Fib-cKO mice with the MMTV-PyMT transgenic mice, a mouse model of breast cancer that mirrors the multistep progression of human breast cancers^23^, and generated female cohorts with the genotypes of MMTV-PyMT;ZEB1^fl/fl^ (designed PyMT-Fib-WT mice), MMTV-PyMT;FSP1-Cre;ZEB1^fl/wt^ (PyMT-Fib-Het mice) and MMTV-PyMT;FSP1-Cre;ZEB1^fl/fl^ (PyMT-Fib-cKO mice) (Fig. 2a). Conditional deletion of ZEB1 protein in the stroma of PyMT-Fib-cKO mammary tumours was confirmed by immunohistochemistry (Fig. 2b). In PyMT-Fib-cKO-ROSA-YFP reporter mice, ZEB1 protein was completely depleted in YFP^+^ stromal cells (Fig. 2c). Furthermore, we observed that CD31-positive endothelial cells in PyMT-Fib-cKO tumours expressed comparable levels of ZEB1 relative to PyMT-Fib-WT tumours (Supplementary Fig. 2i; left panels), suggesting that FSP1-Cre does not target endothelial cells in our system. Of note, we failed to detect ZEB1 protein in CD45-positive leucocytes (including macrophages) in either PyMT-Fib-cKO or PyMT-Fib-WT tumours (Supplementary Fig. 2i; right panels), suggesting that ZEB1 is essentially absent in leucocytes in the MMTV-PyMT model. Interestingly, we found that stromal CAFs remained non-proliferative state and did not undergo apoptosis, independently of ZEB1 conditional deletion (Supplementary Fig. 2j).

In PyMT-WT mice, mammary tumours occurred as early as 45 days of age, and by day 60, 50% of the mice developed tumours (Fig. 2d). In contrast, tumour initiation was markedly delayed in PyMT-Fib-cKO mice with 50% of the targeted mice developing palpable tumours only after 75 days (Fig. 2d). By 16 weeks of age, whereas PyMT-Fib-WT mice developed multiple tumours, a dramatic reduction in tumour size and number was found in -cKO mice (Fig. 2e). The delayed tumour initiation and progression were further supported by a significantly smaller and fewer neoplastic lesions observed in PyMT-Fib-cKO mammary glands as compared with -WT glands (Fig. 2f, g). Approximately 9 weeks after the initial detection of palpable tumours for both mice, PyMT-Fib-cKO mice developed markedly fewer and smaller metastatic lung nodules compared with PyMT-Fib-WT mice (Fig. 2h, i). However, no histological alterations were observed in metastasised tumours of PyMT-Fib-cKO mice versus PyMT-Fib-WT mice (Fig. 2h, i). The percentages of proliferative cells were comparable in metastatic lung nodules of PyMT-Fib-cKO versus PyMT-Fib-WT mice (Supplementary Fig. 2k, l). Furthermore, circulating nucleated cells (CNCs; including circulating tumour cells) displayed a significant reduction in *PyMT* mRNA level in blood recovered from PyMT-Fib-cKO mice relative to controls (Fig. 2j). The lower *PyMT* mRNA levels indicate less CTCs in blood recovered from PyMT-Fib-cKO mice, as only circulating tumour cells express *PyMT*. Of note, PyMT mRNA levels were comparable in primary tumour cells (PTCs) from PyMT-Fib-cKO mice versus PyMT-cKO mice (Fig. 2j), suggesting that ZEB1 ablation does not affect *PyMT* expression. In line with the findings that ZEB1 is predominantly expressed in stromal cells but largely absent in epithelial cells, conditional deletion of ZEB1 in mammary epithelial cells (MaECs) did not affect either the development of mammary glands (Supplementary Fig. 3a–c) or the initiation or growth of mammary tumours induced by *PyMT* oncogene (Supplementary Fig. 3d–h). Furthermore, ZEB1 deletion in MaECs had no impact on metastatic lung nodule formation or tumour grading (Supplementary Fig. 3i, j). Taken together, our data suggest that conditional deletion of ZEB1 in stromal fibroblasts but not MaECs strongly suppresses the initiation, progression and metastasis of mammary epithelial tumours.

### *ZEB1* loss reduces the recruitment of immune and endothelial cells

**Fig. 3:**
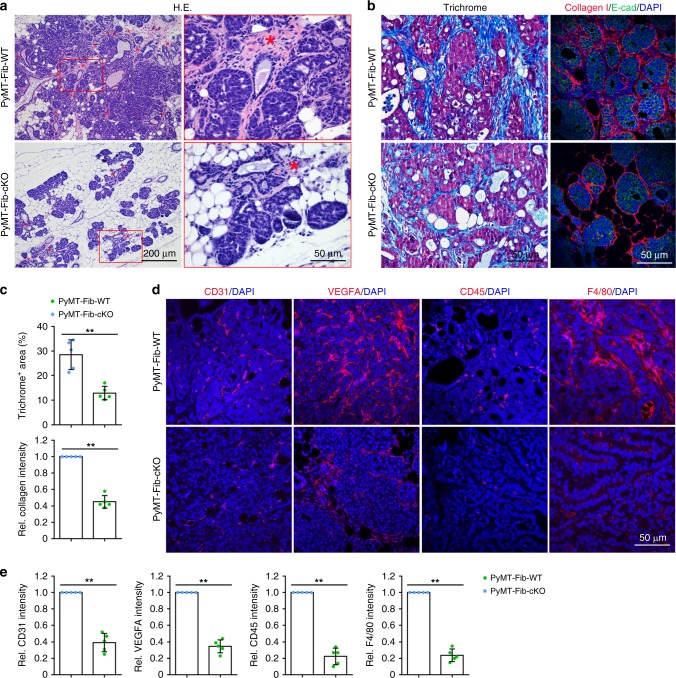
Loss of *ZEB1* in stromal fibroblasts causes impaired ECM deposition and reduced recruitment of immune cells and endothelial cells. **a** Representative images of haematoxylin and eosin (H&E)-stained primary tumours dissected from PyMT-Fib-WT and -cKO females (five mice, each). Magnified areas of boxed sections are shown in right panels. Asterisk denotes the tumour stroma. **b** H&E staining (left) and type I collagen/E-cadherin immunofluorescent staining (right) of primary tumours from PyMT-Fib-WT and -cKO females (images are representative of images from five mice). Nuclei are counterstained with DAPI. **c** Quantification of relative fluorescent intensities in ten random fields of each section of the indicated primary tumours as shown in **b** (*n* = 5 independent experiments). Two-sided Student’s *t* test. **d** Immunofluorescent staining of CD31, VEGFA, CD45 and F4/80 in primary tumours from PyMT-Fib-WT and -cKO mice (images are representative of images from five mice). Nuclei are counterstained with DAPI. **e** Quantification of relative fluorescent intensities in ten random fields of each section of primary tumours as shown in **d** (*n* = 5 independent experiments). Two-sided Student’s *t* test. All data are represented as mean ± s.d. **P < 0.01. The source data are provided as a Source Data file

Histological analyses revealed considerable differences in tumour architecture in PyMT-Fib-WT and PyMT-Fib-cKO tumours. Haematoxylin and eosin (H&E) staining demonstrated that the primary mammary tumours of PyMT-Fib-cKO mice were of a lower grade than tumours from PyMT-Fib-WT mice, and had reduced neoplastic areas in tandem with severely impaired ECM deposition in the stroma (Fig. 3a). Moreover, either Masson’s trichrome staining or immunofluorescent staining of mammary tumour sections indicated significantly reduced deposition of intratumoural type I collagen in the *ZEB1*-deleted stroma (Fig. 3b, c). The impact of inactivation of stromal fibroblast-derived *ZEB1* on the recruitment of cancer-associated immune cells and endothelial cells were assessed as well. A significantly reduced infiltration of CD31-positive endothelial cells was detected in the primary tumours from PyMT-Fib-cKO mice as compared with control littermates (Fig. 3d, e). In support of this finding, expression levels of VEGFA, a potent pro-angiogenic growth factor, were substantially decreased in the tumour stroma of PyMT-cKO mice (Fig. 3d, e). Furthermore, primary tumours of PyMT-Fib-cKO mice exhibited markedly reduced recruitment of CD45-positive leucocytes and F4/80-positive macrophages into the stroma as compared with tumours of PyMT-Fib-WT mice (Fig. 3d, e). From these experiments, we conclude that *ZEB1* ablation in stromal fibroblasts results in impaired ECM deposition and reduced recruitment of cancer-associated immune cells and endothelial cells within mammary tumours.

### *ZEB1* ablation in stromal fibroblasts inhibits the growth and collective invasion of mammary tumour epithelial cells

**Fig. 4:**
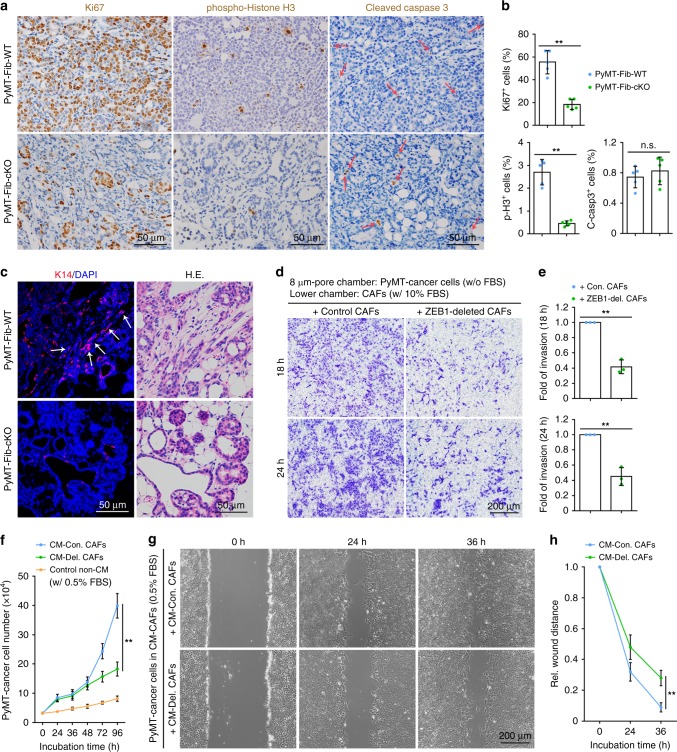
*ZEB1* ablation in stromal fibroblasts reduces mammary tumour epithelial growth and collective invasion. **a** Immunohistochemical analyses of Ki67, phospho-histone H3 and cleaved caspase 3 in advanced-stage primary tumours derived from PyMT-Fib-WT and -cKO mice (images are representative of images from five mice). The arrows in the bottom panels denote cells positive for cleaved caspase 3 (i.e., apoptotic cells). **b** Quantification of Ki67-, phospho-histone H3 (p-H3)- and cleaved caspase 3 (C-casp3)-positive cells in the indicated primary tumours as described in **a**. For quantification, around 1000 cells are counted in ten random fields of each section (*n* = 5 independent experiments). **c** K14 immunofluorescent staining of primary tumours from the indicated mice as shown in **a** (images are representative of images from five mice). Haematoxylin and eosin (H&E)-stained images are shown in the right panels. Nuclei are DAPI-stained. The arrows mark strands of K14^+^ invading leader cells at the invasive borders. **d** Boyden chamber invasion assay of PyMT-cancer cells. Invaded cancer cells on the lower surface of transwell inserts were fixed, stained with crystal violet and counted (representative images are from three independent experiments). **e** Quantification of invaded cells as described in **d** (*n* = 3 independent experiments). Two-sided Student’s *t* test. **f** Proliferation assay of PyMT-cancer cells. Cancer cells were seeded in control non-CM (containing 0.5% FBS) or CM (containing 0.5% FBS) generated from control and ZEB1-deleted CAFs, and cultured for the indicated hours. Cells were harvested and counted under a microscope (*n* = 3 independent experiments). CM conditioned medium. Two-sided Student’s *t* test. **g**, **h** Migration wound-healing assay of PyMT-cancer cells cultured in the conditioned medium generated from control and ZEB1-deleted CAFs (representative images are from three independent experiments). Quantification of wound healing is shown in **h** (*n* = 3 independent experiments). All data are represented as mean ± s.d. **P < 0.01, n.s. not significant. The source data are provided as a Source Data file

Because the deposition of ECM and the recruitment of immune cells as well as endothelial cells are critical steps during tumour progression^7–12^, we assessed the impact of ablation of stromal fibroblast-derived *ZEB1* on the growth and invasion of mammary tumour epithelial cells in vivo and in vitro. A marked reduction in numbers of proliferative and mitotic cells was observed in PyMT-Fib-cKO mammary tumours at the early- and advanced stages, whereas numbers of pro-apoptotic cells were comparable in PyMT-Fib-WT and PyMT-Fib-cKO mammary tumours at both stages (Fig. 4a, b; Supplementary Fig. 4a, b). Cytokeratin-14 (K14) marks a subpopulation of highly migratory carcinoma cells that are capable of initiating collective invasion, a critical step in metastatic progression of malignant mammary tumours^29,30^. We found that K14-positive cells were enriched at the invasive border in PyMT-Fib-WT mammary tumours, forming strands that invade into the surrounding stromal tissues (Fig. 4c). In contrast, PyMT-Fib-cKO mammary tumours failed to exhibit a locally invasive phenotype, and the invasive strands were almost completely absent in the stroma (Fig. 4c).

We next evaluated whether *ZEB1* deletion in stromal fibroblasts could affect their crosstalk with the PyMT-induced breast cancer cells, which in turn impairs their abilities to promote the in vitro migration and invasion of cancer cells. To do this, PyMT-cancer cells (a cell line that was previously established in our laboratory^29^) were cultured in medium without foetal bovine serum (FBS) in the upper chambers of transwell inserts with an 8 -μm pore size that were pre-coated with diluted Matrigel, while mammary stromal ZEB1^fl/fl^ CAFs were seeded in the lower chambers and cultured in medium supplemented with 10% FBS. Remarkably, we found that the invasion of PyMT-cancer cells was significantly inhibited in the presence of ZEB1-deleted CAFs (i.e., adeno-Cre-infected cells) compared with control CAFs (i.e., adeno-βGal-infected cells) at 18 and 24 h of culture (Fig. 4d, e). To test whether this reduced invasion of PyMT-cancer cells was due to the proliferation deficiency in the cells, PyMT-cancer cells were placed in the upper chambers of transwell inserts with a 1 -μm pore size (instead of the 8 -μm pores used in invasion assays). Under these conditions, cells can proliferate but cannot invade into the bottom chamber. We observed that PyMT-cancer cells grew at a comparable rate in the presence of control and ZEB1-deleted CAFs at 18, 24 and 48 h of culture (Supplementary Fig. 4c), suggesting that the defect in cell invasion was not due to proliferation deficiency in the cells at least within 48 h of culture. Furthermore, we assessed the proliferation of PyMT-cancer cells in the presence of control non-conditioned medium or conditioned medium from control CAFs and ZEB1-deleted CAFs (all media were supplemented with 0.5% FBS). We observed that cells grew very slowly in the presence of control non-conditioned medium (Fig. 4f). However, cell growth was significantly increased in the presence of conditioned medium from control CAFs relative to the conditioned medium generated from ZEB1-deleted CAFs at 72 and 96 h of culture (Fig. 4f). Cell proliferation rate was comparable in the presence of conditioned medium from control and ZEB1-deleted CAFs within 48 h of culture (Fig. 4f). In addition, using a migration wound-healing assay, we observed that the migration of PyMT-cancer cells was substantially reduced in the presence of the conditioned medium generated from ZEB1-deleted CAFs relative to the conditioned medium from control CAFs at 36 h of culture (Fig. 4g, h). Altogether, these results suggest that stromal CAFs directly regulate the growth of PyMT-cancer cells (in a paracrine fashion), and *ZEB1* deletion in stromal CAFs strongly suppressed the growth and collective invasion of cancer cells in the PyMT mouse model.

### *ZEB1*-deleted mammary glands have a suppressive tumour microenvironment

**Fig. 5:**
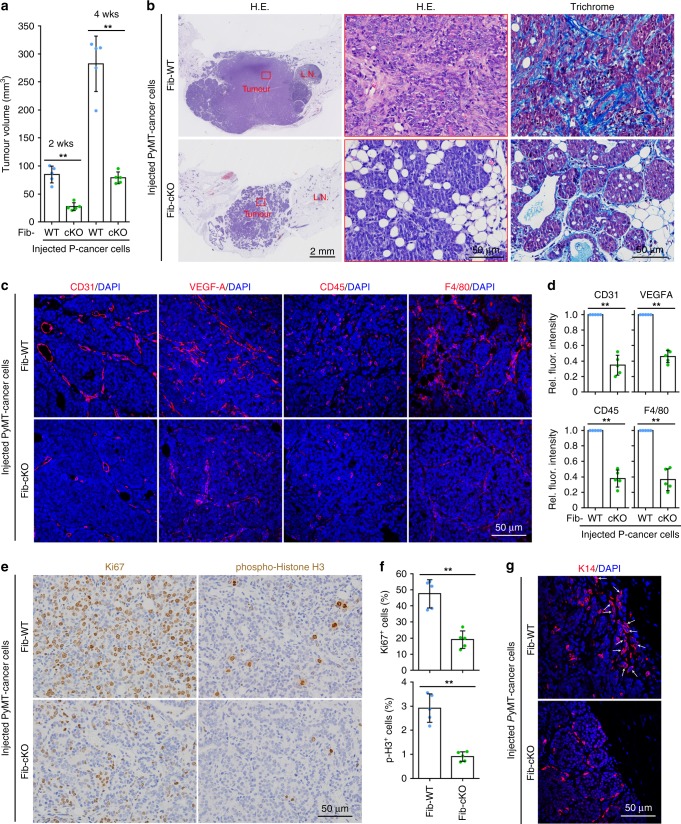
Stroma *ZEB1*-deleted mammary glands exhibit a suppressive tumour microenvironment phenotype. **a** In total, 2 × 10^6^ PyMT-cancer cells were orthotopically transplanted into Fib-WT and -cKO mammary glands (five mice, each), and tumour volume monitored 2 weeks and 4 weeks after transplantation (*n* = 5 independent experiments). Two-sided Student’s *t* test. **b** Haematoxylin and eosin (H&E) and Masson’s staining of orthotopic tumours as described in **a** (images are representative of images from five mice). Magnified areas of boxed sections are shown in middle panels. LN lymph node. **c** Immunofluorescence staining of CD31, VEGFA, CD45 and F4/80 in the indicated orthotopic tumours as described in **a** (images are representative of images from five mice). Nuclei are DAPI stained. **d** Quantification of relative fluorescent intensities in ten random fields of each section of the indicated orthotopic tumours as shown in **c** (*n* = 5 independent experiments). Two-sided Student’s *t* test. **e** Immunohistochemical staining of Ki67 and phospho-histone H3 in the indicated orthotopic tumours as described in **a** (images are representative of images from five mice). **f** Quantification of Ki67- and phospho-histone H3 (p-H3)-positive cells as shown in **e**. For quantification, around 1000 cells are counted in ten random fields of each section (*n* = 5 independent experiments). Two-sided Student’s *t* test. **g** K14 immunofluorescent staining of the indicated orthotopic tumours from the indicated mice, as described in **a** (images are representative of images from five mice). Nuclei are DAPI stained. The arrows denote strands of K14^+^ invading leader cells at the invasive borders. All data are represented as mean ± s.d. **P < 0.01. The source data are provided as a Source Data file

To precisely assess the impact of stromal fibroblast-derived *ZEB1* on the growth and invasion of mammary epithelial tumours in vivo, we orthotopically injected 2 × 10^6^ PyMT-cancer cells into the fat pads of Fib-WT and Fib-cKO mice with tumour growth and invasion monitored. As shown, the growth of orthotopic tumours formed in Fib-cKO glands was remarkably reduced at 2 weeks and 4 weeks (end point) post transplantation relative to tumours retrieved from Fib-WT glands (Fig. 5a). The orthotopic tumours formed in Fib-cKO glands exhibited a more differentiated phenotype in tandem with a markedly decreased deposition of intratumoural type I collagen compared with tumours in Fib-WT glands (Fig. 5b; Supplementary Fig. 5). A markedly reduced recruitment of endothelial cells, leucocytes and macrophages was observed in the orthotopic tumours formed in Fib-cKO mammary glands relative to tumours formed in Fib-WT glands (Fig. 5c, d). Expression levels of VEGFA were also found to be downregulated in the tumours formed in Fib-cKO glands relative to Fib-WT glands (Fig. 5c, d). Furthermore, the orthotopic tumours formed in Fib-cKO mammary glands showed a robust decrease in the percentage of proliferative and mitotic cells in tandem with a markedly reduced enrichment of K14-positive cells at the invasive border (Fig. 5e–g). These results demonstrated that stroma *ZEB1*-deleted mammary glands exhibit a suppressive tumour microenvironment phenotype, thereby resulting in impaired tumour growth and invasion within the conditionally targeted mammary glands.

### ZEB1 functions as a p53 inhibitor in stromal CAFs

**Fig. 6:**
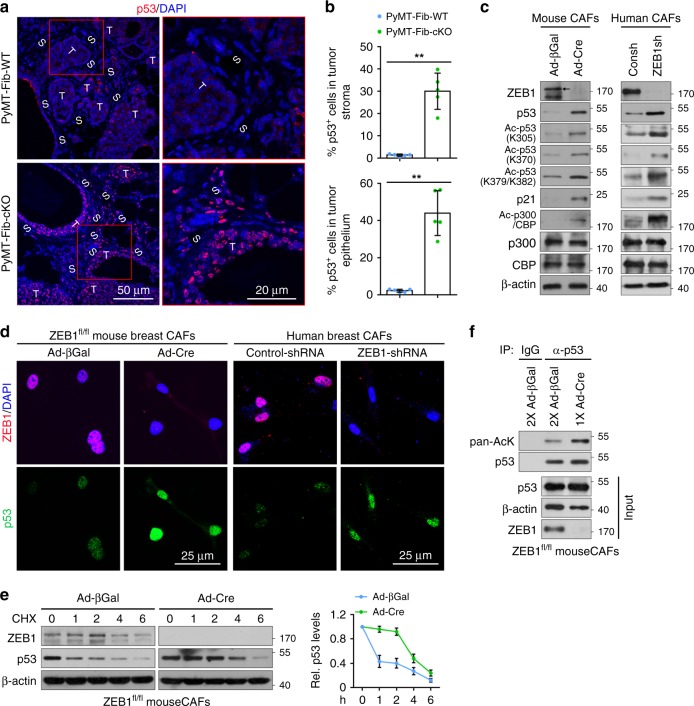
ZEB1 functions as a p53 inhibitor in mammary stromal CAFs. **a** Immunofluorescence staining of p53 in PyMT-Fib-WT and -cKO tumours (images are representative of images from five mice). Magnified areas of boxed sections are shown in right panels. Nuclei are DAPI stained. **b** Quantification of p53-positive cells in stromal and epithelial compartments of the indicated primary tumours. Around 1000 cells are counted in ten random fields of each section (*n* = 5 independent experiments). Two-sided Student’s *t* test. **c** Immunoblot (IB) analysis of primarily cultured control and ZEB1-deleted mouse (left panels) and human (right panels) breast CAFs using the indicated antibodies. **d** Immunofluorescence staining of ZEB1 and p53 in the indicated CAFs as described in **c** (representative images are from three independent experiments). Nuclei are DAPI stained. **e** Cycloheximide (CHX, 100 μg/ml) pulse-chase analysis of p53 protein levels in control and ZEB1-deleted CAFs. Quantification of p53 protein expression levels are shown in right panels (*n* = 3 independent experiments). **f** Lysates from control and ZEB1-deleted CAFs loaded at a ratio of 2:1 were subjected to immunoprecipitation (IP) and IB assays. Arrows mark-specific bands with the expected molecular weights. All representative blots shown are from three independent experiments. All data are represented as mean ± s.d. **P < 0.01. Unprocessed original scans of blots are shown in Supplementary Fig. 9. The source data are provided as a Source Data file

Because the tumour suppressor p53 has been reported to play a critical role in controlling stromal and carcinoma cells expansion during tumour initiation and progression^31,32^, we sought to determine whether ZEB1 deletion could affect the p53 expression level in stromal CAFs. Immunofluorescence analysis illustrated that p53 protein was barely detected in PyMT-Fib-WT mammary tumours, whereas its levels were dramatically increased in both stromal and epithelial compartments of PyMT-Fib-cKO tumours (Fig. 6a, b). In vitro, protein levels of p53 and its downstream target p21 were both increased in ZEB1-deleted mouse CAFs (i.e., adeno-Cre infected cells) isolated from PyMT;ZEB1^fl/fl^ mammary tumours, recapitulating the in vivo phenotype (Fig. 6c, d; left panels). Notably, p53 and p21 protein levels were likewise upregulated in ZEB1-silenced human CAFs (i.e., ZEB1-shRNA infected cells) that were freshly isolated from breast cancer patient samples (Fig. 6c, d; right panels). Although ZEB1 did not affect the mRNA expression of *p53*, deletion of ZEB1 in CAFs markedly increased *p21* mRNA levels while remarkably decreasing expression of *survivin*, a p53-repressed gene^33^ (Supplementary Fig. 6a). Interestingly, both *ZEB2* and *Snail1* mRNA levels dropped markedly following ZEB1 deletion (Supplementary Fig. 6a). Furthermore, ZEB1 deletion in CAFs increased expression levels of phospho-p53 at S15 and S392, but did not affect the levels of γ-H2AX and cleaved caspase 3, suggesting that deletion of ZEB1 in CAFs stabilises p53 protein (see below), but does not cause DNA damage or induce apoptosis (Supplementary Fig. 6b). Concurrently, deletion of ZEB1 in CAFs increased p53 half-life while reducing p53 ubiquitination (Fig. 6e; Supplementary Fig. 6c), suggesting that ZEB1 regulates p53 protein expression without affecting its transcription. Of note, increases in p53 protein levels are associated with increased levels of p53 acetylation^29,34^, and following ZEB1 deletion, p53 acetylation (K305, K370, K379 in mouse and K382 in human) levels increased (Fig. 6c). Given that p53 is acetylated at multiple sites that can each play critical roles in controlling its protein stability and transcriptional activity^29,34^, total acetylation levels of p53 were assessed by immunoprecipitating analysis of lysates from control or ZEB1-deleted CAFs (containing equalised mounts of p53) with antibodies directed against pan-acetylated lysine (pan-Ack) or p53. As indicated, ZEB1-deleted CAFs displayed a robustly increased percentage of acetylated p53 molecules (Fig. 6f). Immunofluorescence analysis further demonstrated that the levels of co-localised pan-AcK and p53 were remarkably increased in ZEB1-deleted CAFs (Supplementary Fig. 6d). Of note, the pan-AcK signal almost completely disappeared in p53-silenced CAFs, verifying the specificity of the Pan-AcK (Supplementary Fig. 6e). Importantly, the ability of ZEB1 to regulate acetylation-linked events is not limited to p53. Without affecting total levels of p300 and CBP, two transcriptional coactivators for p53 acetylation^35,36^, deletion of ZEB1 in mouse and human breast CAFs upregulated the autocatalytic acetylation of p300/CBP (K1499 in p300 and K1535 in CBP) (Fig. 6c). Treatment of CAFs with the p300/CBP pharmacological inhibitor, C646, efficiently blocked ZEB1-regulated acetylation of p53 or the expression of p53 and p21 (Supplementary Fig. 6f).

**Fig. 7:**
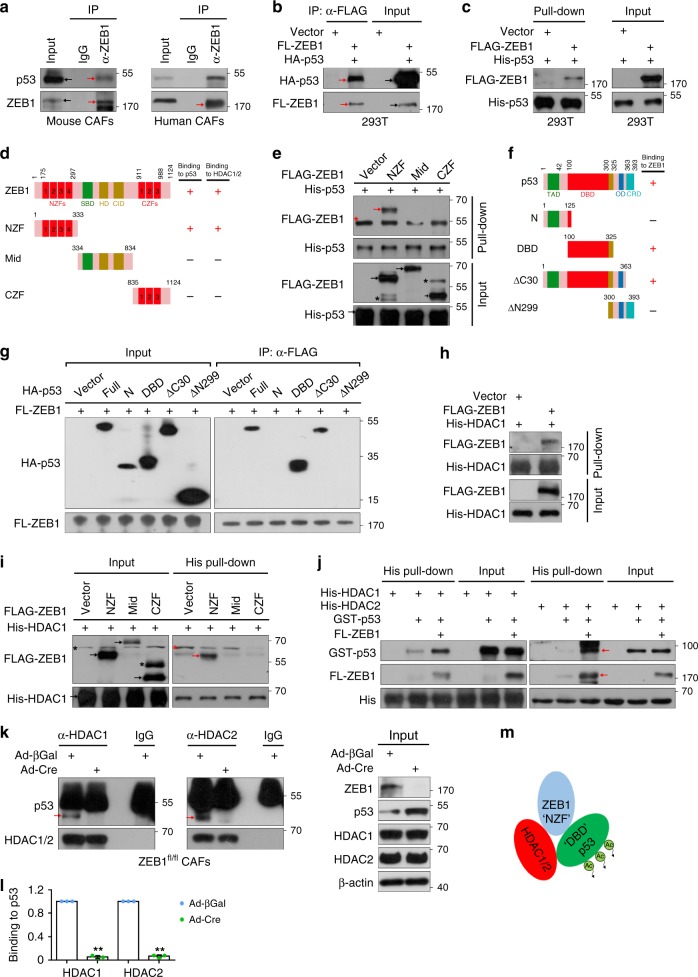
ZEB1 interacts with p53 and HDAC1/2 to form a trimetric complex. **a** Lysates from mouse (left) and human (right) stromal CAFs were subjected to co-IP assay. **b** Lysates from 293T cells that were transfected with FLAG-ZEB1 and HA-p53 were subjected to IP assay. **c** Recombinant His-p53 protein bound to Ni-NTA beads was co-incubated with FLAG or FLAG-ZEB1 protein (FLAG peptide eluted), and the mixture was subjected to His pull-down assays. **d** Schematic representation of ZEB1 fragmental proteins binding to p53, HDAC1 and HDAC2 proteins. NZF N-terminal zinc-finger, CZF C-terminal zinc-finger, SBD Smad-binding domain, HD homeodomain, CID CtBP interaction domain. **e** Pull-down assay of His-p53 by Ni-NTA and co-IP of the indicated ZEB1 fragmental proteins. **f** Schematic representation of p53 fragmental proteins binding to ZEB1 protein. TAD transcription-activation domain, DBD DNA-binding domain, OD homo-oligomerization domain, CRD C-terminal regulatory domain. **g** 293T cells were transfected with FLAG-ZEB1 and HA-p53 fragments, and the cell lysates were subjected to co-IP assays. **h**, **i** Recombinant His-HDAC1 protein bound to Ni-NTA beads was co-incubated with FLAG-ZEB1 (**h**) or FLAG-ZEB1 fragments (**i**), and the mixture was subjected to His pull-down assays. **j** Recombinant His-HDAC1 or His-HDAC2 proteins bound to Ni-NTA beads was co-incubated with GST-p53 protein (eluted) and FLAG-ZEB1 (FLAG peptide eluted), and the mixture was subjected to His pull-down assays. **k**, **l** Lysates from control and ZEB1-deleted CAFs were subjected to co-IP assays using anti-HDAC1/2 antibodies (**k**). Quantification of binding of p53 to HDAC1/2 (**l**) (*n* = 3 independent experiments). Two-sided Student’s *t* test. **m** A schematic representation of the ZEB1-p53-HDAC1/2 tri-molecular complex with the binding domains responsible for the interaction of ZEB1-p53 and ZEB1-HDAC1/2. Asterisks and arrows mark non-specific bands and specific bands with the expected molecular weights, respectively. All representative blots shown are from three independent experiments. All data are represented as mean ± s.d. **P < 0.01. Unprocessed original scans of blots are shown in Supplementary Fig. 9. The source data are provided as a Source Data file

Consistent with an effect of ZEB1 on p53 acetylation, p53/ZEB1 complexes were found in mouse and human breast CAFs with direct binding interactions confirmed using recombinant p53 and ZEB1 (Fig. 7a–c; Supplementary Fig. 6g). To further narrow down the interaction domains of ZEB1, three non-overlapping fragments that encompass N-terminal zinc-finger (NZF) domain, middle domain (Mid) and C-terminal zinc-finger (CZF) domain were constructed and subjected to His pull-down analysis (Fig. 7d). As indicated, NZF domain of ZEB1 was identified as the dominant p53-binding motif (Fig. 7e). In complementary studies using p53 deletion mutants, ZEB1 interacts primarily with the DNA-binding domain (DBD) of p53 (Fig. 7f, g). Earlier studies described that histone deacetylases HDAC1 and HDAC2 can directly or indirectly bind to p53 to form a multi-molecular complex, thereby catalysing p53 deacetylation and promoting its degradation^29,37–39^. We therefore sought to determine whether ZEB1 could bind to HDAC1 and HDAC2. As shown, NZF domain of ZEB1 was identified as the primary HDAC1/2-binding motif (Fig. 7d–i; Supplementary Fig. 6h). We further tested whether ZEB1, p53 and HDAC1/2 proteins could form a trimetric complex. To do this, we purified His-HDAC1/2 (both were beads bound) and GST-p53 (eluted) in *E. coli* bacteria, purified FLAG-ZEB1 protein (FLAG peptide eluted) in 293T cells, incubated these proteins overnight, and performed His pull-down assay. We observed a weak binding between HDAC1 (or HDAC2) and p53 (HDAC2-p53 binding was slightly weaker than HDAC1-p53) (Fig. 7j). However, the binding interactions were significantly increased in the presence of ZEB1 (Fig. 7j), suggesting that the three proteins could form a trimetric complex in vitro. Importantly, the ability of ZEB1 to act as a bridging molecule between endogenous HDAC1/2 and p53 in the trimetric complex was confirmed in co-immunoprecipitation (Co-IP) assays wherein ZEB1 deletion in CAFs almost completely abolished the recruitment of HDAC1/2 to p53 (Fig. 7k–m). Of note, p53 levels were substantially reduced in HDAC1 or HDAC2 IP products recovered from p53-depleted CAFs (Supplementary Fig. 6i).

### The ZEB1/p53 axis regulates tumour growth and progression in a paracrine fashion

**Fig. 8:**
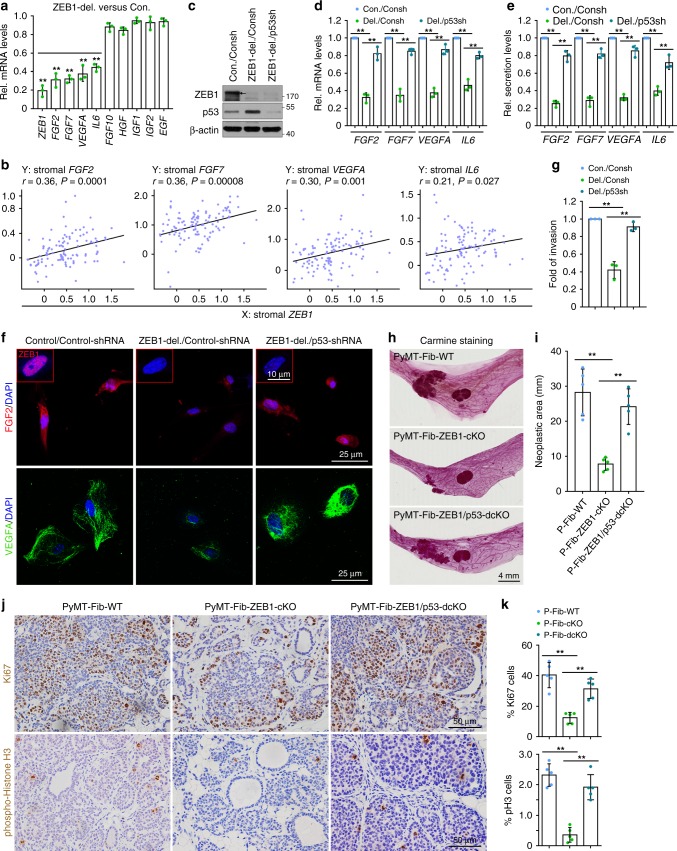
The ZEB1/p53 axis in stromal CAFs controls mammary tumour growth and progression in a paracrine fashion. **a** RT-qPCR analysis of the expressions of the indicated genes in control and ZEB1-deleted CAFs (*n* = 3 independent experiments). Two-sided Student’s *t* test. **b** Positive correlation between the levels of *ZEB1* and p53 targets in the tumour stroma of human breast cancer samples (111 cases) collected from a public data set of gene expression in tumour stroma in breast cancer (GSE9014). *r*: Pearson correlation coefficient. **c** Immunoblot analysis of primarily cultured Con/Consh (i.e., Adeno-βGal plus Lenti-pLKO.1), ZEB1-del./Consh (i.e., Adeno-Cre plus Lenti-pLKO.1) and ZEB1-del./Consh (i.e., Adeno-Cre plus Lenti-pLKO.1-p53shRNA) CAFs. The results are representative of three independent experiments. **d** RT-qPCR analysis of the expressions of the indicated genes in the indicated CAFs as described in **c** (*n* = 3 independent experiments). Two-way ANOVA test. **e** Enzyme-linked immunosorbent assay (ELISA) analysis of the indicated secreted proteins in the indicated CAFs as described in **c** (*n* = 3 independent experiments). Two-way ANOVA test. **f** Immunofluorescence staining of ZEB1 (insets in top panels), FGF2 and VEGFA in the indicated CAFs as described in **c** (representative images are from three independent experiments). **g** Boyden chamber invasion assay of PyMT-cancer cells. Invaded cancer cells on the lower surface were fixed, stained with crystal violet and counted (*n* = 3 independent experiments). Two-way ANOVA test. **h** Whole-mount Carmine red staining of neoplastic mammary glands from PyMT-Fib-WT, -ZEB1-cKO and -ZEB1/p53-dcKO mice (images are representative of images from five mice). **i** Quantification of neoplastic areas in the mammary glands as described in **h** (*n* = 5 independent experiments). Two-way ANOVA test. **j** Immunohistochemical analyses of Ki67 and phospho-histone H3 in primary tumours derived from the indicated mice (images are representative of images from five mice). **k** Quantification of Ki67- and phospho-histone H3 (p-H3)-positive cells as described in **j**. Around 1000 cells are counted in ten random fields of each section (*n* = 5 independent experiments). Two-way ANOVA test. All data are represented as mean ± s.d. **P < 0.01. Unprocessed original scans of blots are shown in Supplementary Fig. 9. The source data are provided as a Source Data file

The stromal fibroblast-derived growth factors, implicated as autocrine and paracrine mediators of stromal–epithelial interactions, are known to play essential roles in tumour initiation and progression^7,12^. Herein, we demonstrated that ZEB1-deleted CAFs markedly reduced transcript levels of various growth factors, including fibroblast growth factor basic (*FGF2*), *FGF7*, vascular endothelial growth factor A (*VEGFA*) and interleukin 6 (*IL6*), but had no effect on expression of other growth factors, such as *FGF10*, hepatocyte growth factor (*HGF*), insulin-like growth factor 1 (*IGF1*), *IGF2* or epidermal growth factor (*EGF*) (Fig. 8a). Of note, p53 has been reported to directly bind to the *FGF2*, *VEGFA* and *IL6* promoters, thereby suppressing their transcription^40–42^. Using chromatin immunoprecipitation (ChIP) assays, we confirmed the loading of p53 onto the proximal but not distal regions of *FGF2*, *FGF7*, *VEGFA* and *IL6* promoters in CAFs, and further showed that ZEB1-deleted CAFs remarkably increased the loading of p53 onto the proximal promoters of these genes (Supplementary Fig. 7a, b). Beyond the transcriptional level, the protein expression and secretion of these signalling molecules were also reduced in ZEB1-deleted CAFs compared with control cells, as assessed by immunofluorescence analysis and enzyme-linked immunosorbent assays (ELISAs), respectively (Supplementary Fig. 7c, d). Importantly, further bioinformatic assays of a public human breast cancer data set (GSE9014) of stromal gene expression revealed a statistically significant correlation between the transcript levels of *ZEB1* and these *TP53* targeting genes (i.e*., FGF2*, *FGF7*, *VEGFA* and *IL6*) in the tumour stroma (Fig. 8b), emphasising the clinical relevance of the ZEB1-TP53 connection in breast tumour stroma. To determine the degree to which the indicated p53 targeting genes serve as the downstream executors of ZEB1, we assessed the impact of p53 silencing on their expression and secretion in ZEB1-deleted CAFs. Immunoblot analysis confirmed increased levels of p53 in ZEB1-deleted CAFs with p53 expression largely abolished following ZEB1/p53 knockdown (Fig. 8c). Importantly, while ZEB1 deletion strongly repressed expression (at both mRNA and protein levels) and secretion of the indicated growth factors, their expression and secretion were partially, but significantly, recovered by decreasing p53 levels (Fig. 8d–f). Consistent with this observation, ZEB1-deleted CAFs markedly reduced the invasiveness index of *PyMT*-cancer cells, and the impaired invasion potential of the cells was completely recovered following p53 knockdown in ZEB1-deleted CAFs (Fig. 8g).

To directly assess the importance of stromal fibroblast-derived p53 as a ZEB1 target during mammary tumour growth and progression in vivo, we generated female cohorts with the genotypes of MMTV-PyMT;ZEB1^fl/fl^;p53^wt/wt^ (PyMT-Fib-WT mice), MMTV-PyMT;FSP1-Cre;ZEB1^fl/fl^;p53^wt/wt^ (PyMT-Fib-ZEB1-cKO) and MMTV-PyMT;FSP1-Cre;ZEB1^fl/fl^;p53^fl/fl^ (PyMT-Fib-ZEB1/p53-dcKO). As expected, p53 expression levels were markedly increased in stromal and epithelial compartments of PyMT-Fib-ZEB1-cKO tumours relative to PyMT-Fib-WT tumours (Supplementary Fig. 7e). Remarkably, p53 in both compartments of PyMT-Fib-ZEB1/p53-dcKO tumours was reduced to levels similar to those found in PyMT-Fib-WT tumours (Supplementary Fig. 7e). In vitro, p53 protein levels were upregulated in PyMT-cancer cells in the presence of the conditioned medium from ZEB1-deleted CAFs relative to the conditioned medium generated from control CAFs, and the upregulated levels of p53 were completely reversed in PyMT-cancer cells in the presence of the conditioned medium generated from ZEB1/p53-deleted CAFs (Supplementary Fig. 7f). The impaired deposition of type I collagen as well as the reduced recruitment of endothelial cells and immune cells observed in PyMT-Fib-ZEB1-cKO tumours were significantly reversed in PyMT-Fib-ZEB1/p53-dcKO tumours (Supplementary Fig. 7g, h). Consistent with these observations, both the markedly reduced growth and proliferative activity of PyMT-Fib-ZEB1-cKO tumours were dramatically reversed following p53 excision (Fig. 8h–k). Taken together, these findings identify p53 as a functionally critical ZEB1 target in vivo, and suggest that the stromal ZEB1/p53 axis plays a critical role in controlling mammary tumour growth and progression in a paracrine fashion.

## Discussion

The accumulated evidence indicates that ZEB1 protein is predominantly present in the stromal compartment, but is largely absent in the epithelial compartment in several types of human cancers, including breast, pancreatic and colorectal cancers^19–22^. In this study, we confirm a similar expressing pattern of ZEB1 (ZEB1) in different molecular subtypes of human and mouse breast cancers, and further show that stromal *ZEB1* levels are markedly elevated upon tumour progression and correlate reversely with the breast cancer patients’ relapse-free survival rates. Of note, we observe significantly upregulated *ZEB1* levels in mammary tumours with abundant stroma, a characteristic indicative of poor patient prognosis^7,43–45^. Having uncovered a significant association between stromal ZEB1 and disease progression in breast cancer patients, we used the MMTV-PyMT breast cancer mouse model as a means to directly dissect the roles for stromal versus epithelial *ZEB1* in mammary tumour progression in vivo. As recently reported^46^, *ZEB1* depletion in pancreatic epithelial cells has no effect on tumour initiation or growth, but reduces tumour grading and metastasis in pancreatic cancer driven by Pdx1-Cre-mediated activation of mutant *Kras* and *p53* (see refs. ^47,48^ for this KPC model). However, here we find that *ZEB1* disruption in MaECs does not affect tumour initiation, growth or metastasis in *PyMT*-induced breast cancer. In striking contrast, *ZEB1* deletion in stromal fibroblasts strongly impairs mammary tumour progression from initiation to metastasis. The considerably variable consequences of *ZEB1* depletion observed in these transgenic lines are probably due to highly differential expression of ZEB1 in the tumour stromal versus epithelial compartments among different cancer types. Nevertheless, our findings suggest that tumour stroma-derived ZEB1 might be implied as a potential prognostic marker for breast cancer, and may be other cancer types.

Stromal CAFs are known to support many different aspects of tumour epithelial progression from initiation to metastasis through production and secretion of growth-stimulatory, pro-angiogenic, immune-modulatory and pro-invasive factors, as well as various ECM components^7–12^. Here, we find that *ZEB1* deletion in stromal CAFs reduces expression and secretion of a variety of paracrine signalling molecules, including FGF2, FGF7, VEGFA and IL6, to the surrounding stroma. These paracrine signalling factors derived from stromal CAFs are shown to bind to specific receptors on the tumour epithelial cells as well as other types of tumour stromal cells such as endothelial cells, leucocytes and macrophages, causing enhanced tumour growth and hastened progression from initiation to metastasis in various types of cancers, including breast cancer^7–9,12,49–53^. Therefore, we propose that *ZEB1* deletion in stromal fibroblasts reduces the secretion of these pro-tumourigenic signalling molecules to the surrounding stroma, thus suppressing mammary tumour growth and progression in a paracrine fashion. The paracrine signalling molecules downregulated in *ZEB1*-deleted stromal CAFs are shown to be direct targets for p53^40–42^, raising the possibility that ZEB1 may function as an upstream regulator of p53 in stromal CAFs. In fact, other zinc-finger transcription factors, including Snail1, Slug and Twist, have been reported to directly or indirectly affect p53 function in vitro and in vivo^29,54–57^. In our working model (Fig. 6s), binding interactions between ZEB1 and p53 in stromal CAFs allow HDAC1/2 to catalyse p53 deacetylation, thereby hastening the functional inactivation of p53 in the cells. Of note, p53 protein levels are likewise increased in PyMT-cancer cells in the presence of the conditioned medium from ZEB1-deleted CAFs compared with control CAFs. These data strongly suggest that stromal CAFs have the ability to directly regulate p53 levels in a paracrine fashion. These data combined with the fact that FGF2 efficiently reduces p53 expression levels while promoting the growth in PyMT-cancer cells (Supplementary Fig. 8a, b; refs. ^31,49^) lead us to propose that ZEB1 deletion in stromal CAFs stabilises p53 protein and thus decreases FGF2 production and secretion in the surrounding stroma (or conditioned medium). In response to lower levels of FGF2, p53 protein levels in PyMT-cancer cells are maintained with attendant effects on epithelial tumour growth and progression^29,56–59^. Importantly, p53 protein levels are largely comparable in epithelial compartment of PyMT-Fib-ZEB1/p53-dcKO versus PyMT-Fib-WT tumours, or in PyMT-cancer cells in the presence of the conditioned medium from ZEB1/p53-deleted CAFs versus control CAFs (Supplementary Fig. 7e, f). Under these conditions, ZEB1/p53-dcKO CAFs retain the ability to express FGF2 (i.e., ZEB1 is no longer required to repress p53), thereby retaining the ability to repress p53 protein levels in PyMT-cancer cells in vivo and in vitro.

In general, our findings identify the ZEB1/p53 axis as a critical stroma-specific signalling pathway that promotes mammary epithelial tumours, and further suggest that genetic or pharmacological inhibition of stromal ZEB1 or ZEB1-p53 interaction could be beneficial in combination with conventional epithelial-targeted therapies.

## Methods

### Mice

Mice were housed under standard specific pathogen-free (SPF) conditions, and all animal experiments were performed in accordance with protocols approved by the Animal Welfare and Ethics Committee of China Pharmaceutical University (AWEC-CPU). The *ZEB1*^floxed/floxed^ *(ZEB1*^fl/fl^) mice were generated in our laboratory. To generate a targeting vector, the initial ~5.6 kb sequences consisting of exon 6 and the upstream 2.1 kb sequences were subcloned and used as long and short homologous arms, respectively, and a flippase recognition target (FRT)-phosphoglycerine kinase (PGK)-neo-FRT-loxP cassette was inserted 367 bp upstream of exon 6 into intronic sequences at position of + 185.4 kb. Similarly, an orphan loxP site was inserted 203 bp downstream of exon 6 at + 187.8 kb. A diphtheria toxin (DTA) cassette was attached to the short-arm side for negative selection. The linearised targeting vector was electroporated into C57BL/6 embryonic stem cells (ESCs), and stable transfected clones were selected with G418 (250 μg/ml). Clones were screened for targeting of the *ZEB1* by southern blotting with *EcoR*V-digested genomic DNA, and recombination was verified at the 5′ end of the construct (three exogenous *EcoR*V sites were located in the FRT-PGKneo-FRT-loxP cassette). Three ESC clones with correct homologous recombination events were used for injection into albino C57BL/6 blastocysts to produce chimeric mice. Chimeric mice were crossed with C57BL/6 mice to confirm transmission of the targeted *ZEB1* allele through the germ line. Chimeras were mated with Flp-deleter mice for excision of the FRT-flanked PGKneo cassette to generate the *ZEB1*^fl^ allele. *ZEB1*^fl/fl^ homozygous conditional knockout mice were born in the expected Mendelian ratios, which indicates that the *ZEB1*^fl^ allele functions equivalently to the wild-type *ZEB1* allele. *ZEB1*^wt/fl^ mice were backcrossed to FBV mice for more than six generations to generate a congenic line before mating with FSP1-Cre, MMTV-Cre and MMTV-PyMT transgenics (all in FVB background). *ZEB1*^fl/fl^ mice will be available to the research community upon reasonable request. MMTV-PyMT (#002374), MMTV-ErbB2/neu (#005038), MMTV-Wnt1 (#002870), p53^fl/fl^ (#008462) and ROSA-YFP (#006148) transgenic mice were purchased from Jackson Laboratory. FSP1-Cre mice were kindly provided by G. Leone^27,28^ (Ohio State University, USA). MMTV-Cre mice (line F, #01XA9) were obtained from US National Cancer Institute (Fredrick, USA). All mice were kept in FVB background with littermates were used in all experiments. The maximal tumour size permitted under the approved protocols is 3 cm (length) × 3 cm (width). The investigators were not blinded to allocation during experiments and outcome assessment. No method of randomisation was used, as mice were segregated into groups based on genotype alone. No statistical method was used to predetermine sample size.

### Tissue arrays

Human breast cancer tissue arrays purchased from US Biomax and Biochain were used in this study to examine ZEB1 protein levels in luminal, HER2^+^ and triple-negative breast tumours with their matched normal breast tissues. The tissue arrays were subjected to standard immunohistochemical analysis as described below.

### Clinical data analyses

The breast tumour stroma microarray gene expression profiles and associated clinical data downloaded from the NCBI gene expression omnibus (GSE9014), the Cancer Genome Atlas (TCGA) RNA-Seq gene expression profiles data for breast cancer from the genomic data commons data portal (https://portal.gdc.cancer.gov/) and the METABRIC microarray gene expression profiles data for breast cancer from cBioPortal (http://www.cbioportal.org). For survival analysis, we compared relapse-free survival time between *ZEB1* higher-expression-level breast cancer patients and *ZEB1* lower-expression-level breast cancer patients. *ZEB1* higher-expression-level and lower-expression-level patients were determined by the median values of *ZEB1* expression. For exploration of the correlation between *ZEB1* gene expression levels and breast cancer tumour stroma levels, we evaluated the level of breast cancer tumour stroma by the tumour stroma score using the ESTIMATE algorithm^60^. For each breast cancer sample, we obtained a tumour stroma score to quantify the level of tumour stroma in the breast cancer tissue. We quantified the correlation of *ZEB1* expression levels with breast cancer tumour stroma levels by the Spearman correlation coefficient (“rho”) of gene expression levels and tumour stroma scores.

### Isolation of stromal CAFs from murine and human mammary tumours

Isolation of luminal epithelial, basal epithelial and stromal cells from mouse mammary tumours was performed as described previously with slight modifications^26,29^. In brief, primary tumours within the fourth glands were dissected, minced into pieces and digested at 37 °C for 1.5 ~2 h in the DMEM medium (Thermo Fisher, #11965–092) supplemented with 5% FBS, 10 ng/ml EGF (Peprotech, #315–09), 500 ng/ml hydrocortisone (Sigma-Aldrich, #H0888), 5 mg/ml insulin (Sigma-Aldrich, #I9278), 20 ng/ml cholera toxin (Sigma-Aldrich, #C8052), 1% penicillin/streptomycin (Thermo Fisher, #15140122) and collagenase/hyaluronidase (STEMCELL Technologies, #07919). Organoids were collected and incubated with 0.25% trypsin-EDTA (Thermo Fisher, #25200–072) for 1.5 min, 5 mg/ml dispase (Thermo Fisher, #17105–041) plus 0.1 mg/ml DNase (Sigma-Aldrich, #900933) for 5 min, and 0.64% NH_4_Cl (STEMCELL Technologies, #07850) for 5 min at 37 °C. Following filtration through a 40 μm cell strainer (BD Biosciences, #352340), cells were harvested and re-suspended in HBSS buffer containing 0.5% BSA. For flow cytometry analysis, single-cell suspensions were incubated with an antibody cocktail containing CD31 (Biolegend, #102504, 1:100), CD45 (Biolegend, #103104, 1:100) and Ter119 (Biolegend, #116204, 1:100), a secondary biotin-labelled antibody cocktail (STEMCELL Technologies, #19153, 1:100), and magnetic beads (STEMCELL Technologies, #19150, 1:200) for 15 min each on ice. The unbound cells were collected, extensively washed and labelled with FITC-CD49f (BD Biosicences, #561893, 1:200) and APC-CD24 (eBioscience, #11–0242–82, 1:1000) for 30 min, followed by DAPI staining for 5 min before FACS analysis. Luminal, basal and stromal cells were sorted based on the profiles of CD31^−^CD45^−^Ter119^−^(LIN^−^)DAPI^−^CD49f^low^CD24^+^, LIN^−^DAPI^−^CD49f^+^CD24^low^ and LIN^−^DAPI^−^CD49f^-^CD24^−^, respectively.

For isolation of human stromal CAFs, freshly isolated breast tumours were rinsed extensively for three times in PBS supplemented with 1% penicillin/streptomycin, chopped into 3 × 3 mm pieces and seeded in culture dishes pre-rinsed with the DMEM medium containing 10% FBS. After 2 h of culture at 37 °C, tumour pieces were fed with the DMEM medium containing 10% FBS, and stromal CAFs migrated from tumour bulks were harvested 4 days later. Normally, mouse and human CAFs were used within five passages. This clinical study was approved by the Ethnic Committee of the First Affiliated Hospital, Nanjing Medical University, and written informed consents were obtained from the patients before procedure.

### Isolation of circulating nucleated cells (CNCs)

Whole blood cells were collected (normally 1.0 ml per mouse), mixed with 0.1 ml of heparin sodium solution (1.1 mg/ml) and centrifuged at 3000*g* for 10 min at room temperature. The pellets were re-suspended in 10 ml of 0.64% NH_4_Cl, incubated for 5 min at 37 °C and centrifuged at 3000*g* for 10 min at room temperature (repeated for three times). Following filtration through a 40 -μm cell strainer, CNCs were harvested for further use.

### Cell culture

HEK293T cells were purchased from ATCC and cultured in the DMEM medium supplemented with 10% FBS and 1% pen/strep. PyMT-cancer cell line was generated in our laboratory as described elsewhere^29^ and cultured in the DMEM/F12 medium containing 5% FBS, 10 ng/ml EGF, 500 ng/ml hydrocortisone, 5 mg/ml insulin, 20 ng/ml cholera toxin, 1% pen/strep. Stromal CAFs were cultured in the DMEM medium supplemented with 10% FBS and 1% pen/strep. Cells were tested for mycoplasma contamination every 2 months, and only mycoplasma-negative cells were used. No cell lines in this study were authenticated in our laboratory. No cell lines used in this study were found in the database of commonly misidentified cell lines that is maintained by ICLAC and NCBI Biosample.

### Orthotopic mammary gland transplantation

Eight-week-old female littermates with genotypes of Fib-WT and Fib-cKO were anaesthetised and injected with 2 × 10^6^ of PyMT-cancer cells at the fourth inguinal mammary glands. Tumour volumes were measured at 2 weeks and 4 weeks post transplantation. Mice were killed 4 weeks after injection, and orthotopic tumours were harvested for further use.

### Histological and immuohistochemical analyses

For whole-mount staining, the fourth inguinal mammary glands were harvested and fixed in Carnoy’s solution (60% ethanol, 30% chloroform and 10% glacial acetic acid, in volume) for 4 h at 4 °C, followed by hydration and carmine red staining. The stained glands were then flattened, dehydrated and mounted. For histological assays, tissues were fixed in 4% paraformaldehyde (PFA) and embedded in paraffin. The embedded tissues were sectioned at 5 μm, deparaffinized and subjected to H&E staining or Masson’s trichrome staining using a kit (Sigma-Aldrich, #HT15) according to the manufacturer’s instruction. For immunohistochemical analysis, deparaffinized sections were rehydrated and subjected to antigen heat retrieval with Antigen Unmasking Solution, citric acid based (pH 6.0; Vector Laboratories, #H-3300). The sections were incubated 0.3% H_2_O_2_ (in PBS) for 20 min at room temperature and then in blocking buffer (5% goat serum in PBS) for 1 h at room temperature. The sections were then incubated overnight at 4 °C in blocking buffer containing primary antibodies against ZEB1 (Santa Cruz, #sc-25388, 1:400), K14 (Biolegend, #905304, 1:200), Ki67 (Abcam, #ab15580, 1:2,000), p-H3 (Cell Signaling, #9701, 1:500) or cleaved-caspase 3 (Cell Signaling, #9664, 1:100), followed by incubation with biotinylated goat anti-mouse (Vector Laboratories, #BA-9200, 1:200) and goat anti-rabbit (Vector Laboratories, #BA-1000, 1:200) secondary antibodies for 30 min at room temperature. Standard ABC kit (Vector Laboratories, #PK-6101) and DAB (Vector Laboratories, #SK-4105) were used for the detection of HRP activity. Slides were counterstained with haematoxylin, dehydrated and mounted. For immunofluorescent analysis, sectioned tissues or cultured cells were incubated overnight at 4 °C in blocking buffer containing primary antibodies against ZEB1 (1:400), GFP (Thermo Fisher, #A10262), E-cadherin (BD Biosciences, #610181, 1:1,000), Collagen I (Abcam, #ab34710, 1:400), CD31 (Dianova, #DIA310, 1:100), VEGFA (Abcam, #ab52917, 1:200), CD45 (Biolegend, #103120, 1:100), F4/80 (Thermo Fisher, #14-4801-81, 1:100), K14, p53 (Cell Signaling, #32532, 1:800), FGF2 (Abcam, #ab8880, 1:100), fibronectin (Abcam, #ab45688, 1:300), Ac-p53-K305 (Abcam, #ab109396, 1:400), Ac-p53-K379 (Cell Signaling, #2570, 1:400), Ac-p53-K370 (Abcam, #ab109396, 1:400) or pan-AcK (Cell Signaling, #9441, 1:200), followed by incubation with goat anti-mouse Alexa 594, goat anti-mouse Alexa 647, goat anti-rabbit Alexa 488, and goat anti-rabbit Alexa 555 secondary antibodies (all from Thermo Fisher, 1:300) for 1 h at room temperature. The sections or cells were then counterstained with DAPI, and images were acquired on a Zeiss LSM800 microscope.

### Immunoblot, immunoprecipitation and His pull-down assays

For immunoblot assay, cells were harvested and lysed in RIPA buffer (Thermo Fisher, #89900) containing protease inhibitor cocktail (Thermo Fisher, #87786). The cell lysates were subjected to immunoblot assay using antibodies against ZEB1 (1:1000), p53 (Cell Signaling, #2524, 1:1000), p53 (Cell Signaling, #32532, 1:800), Ac-p53-K379 (1:500), Ac-p53-K370 (1:1000), Ac-p53-K305 (1:1000), Ac-p300-K1499 (Cell Signaling, #4771, 1:500), p21 (Abcam, #ab188224, 1:200), p16 (Thermo Fisher, #MA5-17142, 1:500), β-actin (Sigma-Aldrich, #A5316, 1:10,000), pan-AcK (1:1000), HA (Cell Signaling, #3724, clone C29F4, 1:1000), FLAG (Sigma-Aldrich, #F3165, 1:2000), His (Cell Signaling, #12698, 1:5000), HDAC1 (Cell Signaling, #5356, 1:1000), HDAC1 (Cell Signaling, #2062, 1:1000), HDAC2 (Cell Signaling, #5113, 1:1000) or HDAC2 (Cell Signaling, #57156, 1:1000). For immunoprecipitation (IP) assay, cells were harvested and lysed in IP lysis buffer (50 mM Tris-HCl, 150 mM NaCl, 1 mM EDTA, 1% NP40, pH 7.4) supplemented with protease inhibitor cocktail for 20 min on ice. The cell lysates were sonicated, clarified and incubated with antibodies against p53 (Cell Signaling, #2524, 1:1000), HDAC1 (Cell Signaling, #5356, 1:1000), FLAG or ZEB1, followed by incubation with pre-cleared Protein A/G agarose beads (Santa Cruz, #sc-2003). The immunocomplexes were subjected to immunoblotting using antibodies against pan-AcK, p53 (Cell Signaling, #32532, 1:800), ZEB1, β-actin, HA or FLAG. For His pull-down assay, His-p53, His-HDAC1 and His-HDAC2 recombinant proteins were expressed and purified using a kit (Thermo Fisher, #K95001) according to the manufacturer’s instruction. The beads-bound proteins were incubated with lysates of HEK293T cells that were transfected with FLAG-ZEB1 full-length or FLAG-ZEB1 truncated mutants at 4 °C overnight on a rotator. The beads were collected, extensively washed, eluted and subjected to immunoblot assay using the indicated antibodies.

### In vitro invasion assays

Control and ZEB1-deleted stromal CAFs (4 × 10^4^) were seeded in the DMEM culture medium with 10% FBS in lower chamber of 24-well plates (BD Biosciences, #354480), and allowed to attach for 24 h. Then, PyMT-cancer cells (1 × 10^5^) were placed in the DMEM/F12 culture medium without FBS in the upper chambers of transwell inserts with an 8 -μm pore size that were pre-coated with diluted Matrigel (1:7; BD Biosciences, #356234). PyMT-cancer cells were allowed to invade into the bottom chamber for 18 and 24 h. Non-invading cells in the upper surface were removed and invaded cells on the lower surface were fixed, stained with crystal violet and counted. In some experiments, PyMT-cancer cells were placed in the upper chambers of transwell inserts with a 1 -μm pore size. Under these conditions, cells can proliferate but cannot invade into the bottom chamber.

### Wound-healing assays

PyMT-cancer cells were seeded in a six-well plate and allowed to grow to 80% confluence. The cell monolayer was subsequently scratched with a 200 -μl pipette tip to create a narrow wound-like gap. Cells were washed twice with PBS and fed with conditioned medium containing 0.5% FBS collected from control and ZEB1-deleted stromal CAFs. The plates were photographed at 0, 24 and 36 h, and the wound distances were measured.

### Cell-proliferation assays

PyMT-cancer cells (3 × 10^4^) were cultured in the DMEM medium with 10% FBS for 12 h. Cells were washed twice with PBS, fed with the DMEM medium (containing 0.5% FBS) or conditioned medium (containing 0.5% FBS) generated from control and ZEB1-deleted CAFs, and cultured for additional 24, 36, 48, 72 and 96 h. Cells were harvested and counted under a microscope.

### ELISAs

Conditioned media were collected from control, ZEB1-deleted and ZEB1/p53-deleted stromal CAFs, clarified by centrifugation and frozen for further use. ELISAs were performed using the kits according to the manufacturer’s instruction (R&D Systems, FGF2, #MFB00; VEGFA, #MMV00; IL6, #M6000B).

### Cloning, cell transfection and virus production and infection

Full-length and truncated mutants of ZEB1 were amplified by PCR and subcloned into a p3ˣFLAG-CMV vector (Sigma-Aldrich, #E7533). Full-length cDNAs of p53, HDAC1 and HDAC2 were amplified by PCR and subcloned into a pET23a( + ) vector (Merck, #69745). HA-tagged p53 wild-type and truncated mutants were provided by P.-C. Yang^61^ (Academia Sinica, China). pLKO.1-mp53shRNA was generated in our laboratory as described previously^29^. Production and infection of lentiviral particles were performed as described previously^29^. Stromal CAFs were incubated with a mixture of conditioned medium (containing lentiviral particles) and fresh medium at a ratio of 1:1 for 24 h, and refed with the mixture for another 24 h in the presence of 8 μg/ml polybrene (Sigma-Aldrich, #H9268). Recombinant adenovirus expressing βGal (#CV1001) or Cre (#CV10010) was purchased from Vigene Biosciences. Stromal CAFs isolated from PyMT;ZEB1^fl/fl^ mammary tumours were infected with adeno-βGal or adeno-Cre (MOI = 200) for 24 h, and re-infected for additional 24 h to generate control or ZEB1-deleted cells.

### RT-qPCR assays

The total RNA was isolated and reversely transcribed using the RNeasy kit (Qiagen, #74104) and the PrimeScript RT reagent kit (TaKaRa, #RR037A), respectively, according to the manufacturer’s instructions. qPCR was performed using the SYBR Green PCR Master Mix (Thermo Fisher, #4368706), and relative mRNA expression was normalised to *GAPDH* (primer sequences are shown in Supplementary Table 1).

### Chromatin IP assays

Stromal CAFs were fixed in 1% formaldehyde for 10 min, followed by incubation in 1.25 M glycine for 5 min. Cells were lysed in IP lysis buffer (50 mM Tris-HCl, 150 mM NaCl, 1 mM EDTA, 1% NP40, pH 7.4) supplemented with protease inhibitor cocktail for 20 min on ice. Following optimised sonification, the cell lysates were clarified and precleared with Protein A/G agarose beads and salmon sperm DNA (Thermo Fisher, #15632011) for 1 h at 4 °C. The precleared cell lysates were incubated with the anti-p53 antibody (Cell Signaling, #2524, 1:200) or control mouse IgG1 (Cell Signaling, #5415, 1:200) at 4 °C overnight on a rotator, followed by incubation with precleared Protein A/G agarose beads and salmon sperm DNA (Thermo Fisher, #15632011) for 1 h at 4 °C. The immunocomplexes were sequentially washed with low-salt wash buffer once, high-salt wash buffer once and TE buffer twice and eluted with elution buffer (1% SDS, 0.1 M NaHCO_3_) twice. The eluted DNA–protein complexes were incubated with 0.2 M NaCl overnight at 65 °C, RNase A for 30 min at 37 °C and proteinase K (Thermo Fisher, #AM2548) for 1.5 h at 45 °C. The bound DNA was purified using a kit (Sigma-Aldrich, #NA1020) according to the manufacturer’s instructions, and then subjected to PCR or qPCR analysis (primer sequences are shown in Supplementary Table 1).

### Genotyping assays

Genotyping assays were performed as described previously^29^. Transgenic mouse tails were cut (~2 mm) and digested at 95 °C for 30 min in 50 μL of buffer 1 (containing 10 N NaOH and 0.5 M EDTA, pH 12.0). An equal amount of buffer 2 (containing 40 mM Tris-HCl, pH 5.0) was added to neutralise buffer 1. The mixtures were immediately vortexed and centrifuged at 12,000*g* for 5 min. For genotyping, 1 μL of the supernatants (tail genomic DNA) was used as a template in 20 μL of PCR reaction mixture containing 10 μL 2 × GoTaq GreenMaster Mix (Promega, catalogue No. M7123), 0.5 μL forward/reverse primers and 8.5 μL H_2_O (primer sequences are shown in Supplementary Table 1).

### Statistics

The data were presented as mean ± s.d. Statistical analysis was carried out as described in each corresponding figure legend, and sample sizes are shown in each corresponding figure legend. *P* < 0.05 is considered significant.

### Reporting summary

Further information on research design is available in the Nature Research Reporting Summary linked to this article.

## Supplementary information

Supplementary Information

Reporting Summary

Source Data

## Data Availability

Patient data supporting the analyses in Figs 1c–g, 7b and Supplementary Fig. 1 are accessed from the NCBI gene expression omnibus (GSE9014), *TCGA* RNA-Seq gene expression profiles data for breast cancer (https://portal.gdc.cancer.gov/) and the *METABRIC* microarray gene expression profiles data for breast cancer (http://www.cbioportal.org). The source data underlying Figs. 1b-g, 1j, 2d, 2e, 2g, 2i, 2j, 3c, 3e, 4b, 4e, 4f, 4h, 5a, 5d, 5f, 6b, 6e, 7l, 8a, 8d, 8e, 8g, 8i, 8k and Supplementary Figs. 2e, 2l, 3e, 3h, 3j, 4b, 6a, 7b, 7d, 7h, 8b are provided as a Source Data file. Unprocessed original scans of blots are shown in Supplementary Fig. 9. The remaining data are contained within the article, Supplementary Information or available from the authors upon request. A reporting summary for this article is available as a Supplementary Information file.

## References

[1] Lengauer C, Kinzler KW, Vogelstein B (1998). Genetic instabilities in human cancers. Nature.

[2] Vogelstein B, Kinzler KW (2004). Cancer genes and the pathways they control. Nat. Med..

[3] Hanahan D, Coussens LM (2012). Accessories to the crime: functions of cells recruited to the tumour microenvironment. Cancer Cell.

[4] Hanahan D, Weinberg RA (2011). Hallmarks of cancer: the next generation. Cell.

[5] McAllister SS, Weinberg RA (2014). The tumour-induced systemic environment as a critical regulator of cancer progression and metastasis. Nat. Cell Biol..

[6] Mueller MM, Fusenig NE (2004). Friends or foes-bipolar effects of the tumour stroma in cacner. Nat. Rev. Cancer.

[7] Bhowmick NA, Neilson EG, Moses HL (2004). Stromal fibroblasts in cancer initiation and progression. Nature.

[8] Gascard P, Tlsty TD (2016). Carcinoma-associated fibroblasts: orchestrating the composition of malignancy. Gene Dev..

[9] Kalluri R, Reisberg M (2006). Fibroblasts in cancer. Nat. Rev. Cancer.

[10] Ostman A, Augsten M (2009). Cancer-associated fibroblasts and tumor growth-bystanders turning into key players. Curr. Opin. Genet. Dev..

[11] Pietras K, Ostman A (2010). Hallmarks of cancer: interactions with the tumor stroma. Exp. Cell Res..

[12] Bhowmick NA, Moses HL (2005). Tumor-stroma interactions. Curr. Opin. Genet. Dev..

[13] Spaderna S (2008). The transcriptional repressor ZEB1 promotes metastasis and loss of cell polarity in cancer. Cancer Res..

[14] Wellner U (2009). The EMT-activator ZEB1 promotes tumourigenicity by repressing stemness-inhibiting microRNAs. Nat. Cell Biol..

[15] Zhang P (2014). ATM-mediated stabilization of ZEB1 promotes DNA damage response and radioresistance through CHK1. Nat. Cell Biol..

[16] Vandewalle C, Van Roy F, Berx G (2009). The role of the ZEB1 family of transcription factors in development and disease. Cell. Mol. Life Sci..

[17] Kiweler N (2018). The histone deacetylases HDAC1 and HDAC2 are required for the growth and survival of renal carcinoma cells. Arch. Toxicol..

[18] Lazarova D, Bordonaro M (2017). ZEB1 mediates drug resistance and EMT in p300-deficient CRC. J. Cancer.

[19] Bronsert P (2014). Prognostic significance of zinc-finger E-box binding homeobox 1 (ZEB1) expression in cancer cells and cancer-associated fibroblasts in pancreatic head cancer. Surgery.

[20] Chaffer CL (2013). Poised chromatin at the ZEB1 promoter enables breast cancer cell plasticity and enhances tumorigenicity. Cell.

[21] Soini Y (2011). Transcription factors ZEB1, Twist and Snail1 in breast carcinoma. BMC Cancer.

[22] Spaderna S (2006). A transit, EMT-linked loss of basement membranes indicates metastasis and poor survival in colorectal cancer. Gastroenterology.

[23] Lin EY (2003). Progression to malignancy in the polyoma middle T oncoprotein mouse breast cancer model provides a reliable model for human disease. Am. J. Pathol..

[24] Muller WJ (1988). Single-step induction of mammary adenocarcinoma in transgenic mice bearing the activated c-neu oncogene. Cell.

[25] Herschkowitz JI (2007). Identification of conserved gene expression features between murine mammary carcinoma models and human breast tumors. Genome Biol..

[26] Ye X (2015). Distinct EMT programs control normal mammary stem cells and tumor-initiating cells. Nature.

[27] Trimboli AJ (2008). Direct evidence for epithelial-mesenchymal transitions in breast cancer. Cancer Res..

[28] Trimboli AJ (2009). Pten in stromal fibroblasts suppresses mammary epithelial tumours. Nature.

[29] Ni T (2016). Snail1-dependent p53 repression regulates expansion and activity of tumour-initiating cells in breast cancer. Nat. Cell Biol..

[30] Cheung KJ (2013). Collective invasion in breast cancer requires a conserved basal epithelial program. Cell.

[31] Procopio M-G (2015). Combined CSL and p53 downregulation promotes cancer-associated fibroblast activation. Nat. Cell Biol..

[32] Bar J, Moskovits N, Oren M (2010). Involvement of stromal p53 in tumor-stroma interactions. Semin. Cell Dev. Biol..

[33] Rauch A (2018). Survivin antagonizes chemotherapy-induced cell death of colorectal cancer cells. Oncotarget.

[34] Tang Y (2008). Acetylation is indispensable for p53 activation. Cell.

[35] Thompson PR (2004). Regulation of the p300 HAT domain via a novel activation loop. Nat. Struct. Mol. Biol..

[36] Ferreon JC (2009). Cooperative regulation of p53 by modulation of ternary complex formation with CBP/p300 and HMD2. Proc. Natl Acad. Sci. USA.

[37] Ito A (2002). MDM2-HDAC1-mediated deacetylation of p53 is required for its degradation. EMBO J..

[38] Wagner T (2014). Histone deacetylase 2 controls p53 and is a critical factor in tumorigenesis. Biochim Biophys. Acta.

[39] Juan LJ (2000). Histone deacetylases specifically down-regulate p53-dependent gene activation. J. Biol. Chem..

[40] Ueba T (1994). Transcriptional regulation of basic fibroblast growth factor gene by p53 in human glioblastoma and hepatocellular carcinoma cells. Proc. Natl Acad. Sci. USA.

[41] Pal S, Datta K, Mukhopadhyay D (2001). Central role of p53 on regulation of vascular permeability factor/vascular endothelial growth factor (VPF/VEGF) expression in mammary carcinoma. Cancer Res..

[42] Santhanam U, Ray A, Sehgal PB (1991). Repression of the interleukin 6 gene promoter by p53 and the retinoblastoma susceptibility gene product. Proc. Natl Acad. Sci. USA.

[43] Cardone A (1997). Prognostic value of desmoplastic reaction and lymphocytic infiltration in the management of breast cancer. Panminerva Med..

[44] Ronnov-Jessen L, Petersen OW, Bissel MJ (1996). Cellular changes involved in conversation of normal to malignant breast: importance of the stromal reaction. Physiol. Rev..

[45] Bhowmick NA (2004). TGF-β signaling in fibroblasts modulates the oncogenic potential of adjacent epithelia. Science.

[46] Krebs AM (2017). The EMT-activator ZEB1 is a key factor for cell plasticity and promotes metastasis in pancreatic cancer. Nat. Cell Biol..

[47] Hingorani SR (2005). Trp53R172H and KrasG12D cooperate to promote chromosomal instability and widely metastatic pancreatic ductal adenocarcinoma in mice. Cancer Cell.

[48] Zheng X (2015). Epithelial-to-mesenchymal transition is dispensable for metastasis but induces chemoresistance in pancreatic cancer. Nature.

[49] Giulianelli S (2008). Carcinoma-associated fibroblasts activate progesterone receptors and induce hormonic independent mammary tumor growth: a role for the FGF-2/FGFR-2 axis. Int. J. Cancer.

[50] Kim KJ (1993). Inhibition of vascular endothelial growth factor-induced angiogenesis suppresses tumour growth in vivo. Nature.

[51] Saaristo A, Karpanen T, Alitalo K (2000). Mechanisms of angiogenesis and their use in the inhibition of tumor growth and metastasis. Oncogene.

[52] Guo Y (2012). Interleukin-6 signaling pathway in targeted therapy for cancer. Cancer Treat. Rev..

[53] Schafer ZT, Brugge JS (2007). IL-6 involvement in epithelial cancers. J. Clin. Invest..

[54] Kajita M, McClinic KN, Wade PA (2004). Aberrant expression of the transcription factors Snail and Slug alters the response to genotoxic stress. Mol. Cell. Biol..

[55] Kim J (2014). Cooperative actions of p21WAF1 and p53 induce Slug protein degradation and suppress cell invasion. EMBO Rep..

[56] Piccinin S (2012). A ‘Twist box’ code of p53 inactivation: twist box: p53 interaction promotes p53 degradation. Cancer Cell.

[57] Beck B (2015). Different levels of Twist1 regulate skin tumour initiation, stemness, and progression. Cell Stem Cell.

[58] Godar S (2008). Growth-inhibitory and tumour-suppressive functions of p53 depend on its repression of CD44 expression. Cell.

[59] Chang CJ (2011). p53 regulates epithelial-mesenchymal transition and stem cell properties through modulating miRNAs. Nat. Cell Biol..

[60] Yoshihara K (2013). Inferring tumour purity and stromal and immune cell admixture from expression data. Nat. Commun..

[61] Wang SP (2009). p53 controls cancer cell invasion by inducing the MDM2-mediated degradation of Slug. Nat. Cell Biol..

