# Supplementary material for: A ZEB1/p53 signaling axis in stromal fibroblasts promotes mammary epithelial tumours
Source: Nat Commun. 2019 Jul 19;10:3210. doi: 10.1038/s41467-019-11278-7 (PMC6642263; doi:10.1038/s41467-019-11278-7)
Supplement: Supplementary file 1 — Supplementary Information [file 41467_2019_11278_MOESM1_ESM.pdf]

Supplementary Information

A ZEB1/p53 signaling axis in stromal fibroblasts promotes mammary epithelial tumours

Fu et al.

Supplementary Figures

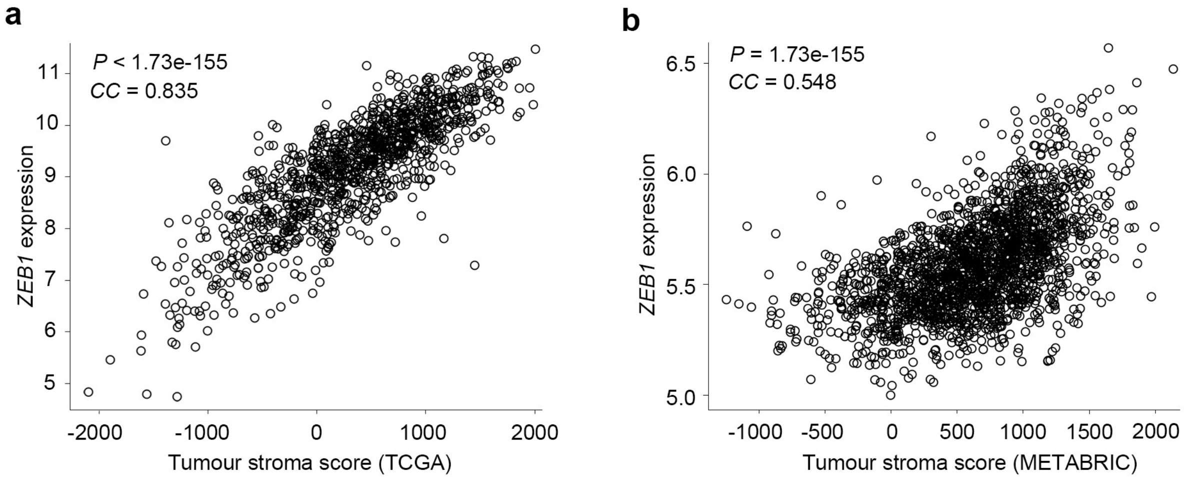

Supplementary Fig. 1 Expression pattern of ZEB1 in mammary tumours. a,**b** A positive correlation between *ZEB1* levels and the tumour stroma scores in breast cancer samples collected from the TCGA (**a**) and METABRIC (**b**) data sets. *CC*: Spearman correlation coefficient.

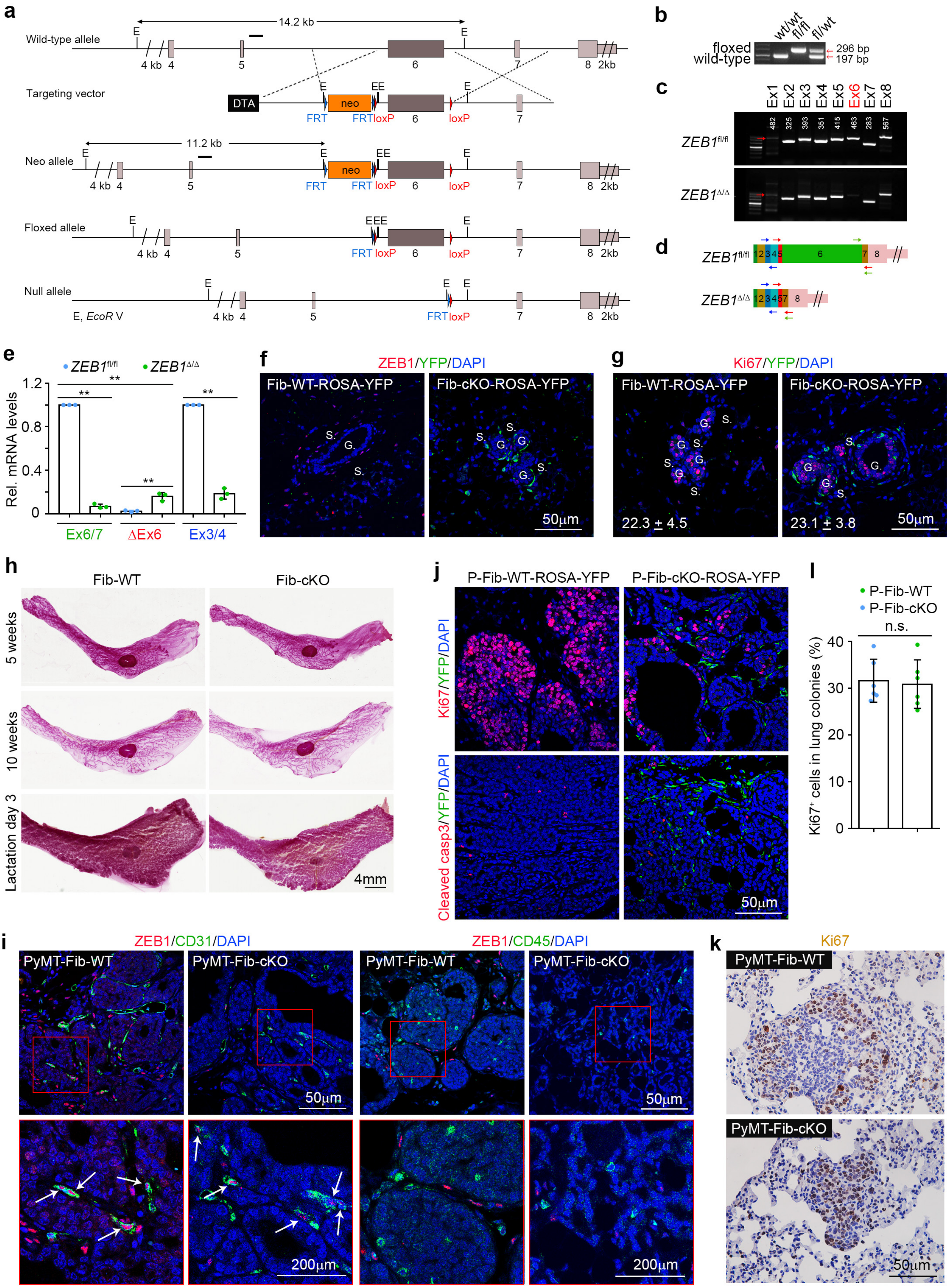

a
Wild-type allele
14.2 kb
E
4 kb 4 5 6 7 8 2kb
Targeting vector
DTA
neo
FRT FRT loxP 6 loxP 7
Neo allele
11.2 kb
Floxed allele
Null allele
E, EcoR V
b
wt/wt fl/fl fl/wt
floxed
wild-type
296 bp
197 bp
c
Ex1 Ex2 Ex3 Ex4 Ex5 Ex6 Ex7 Ex8
482 325 393 351 415 463 283 567
ZEB1fl/fl
ZEB1Δ/Δ
d
ZEB1fl/fl
ZEB1Δ/Δ
e
ZEB1fl/fl ZEB1Δ/Δ
Rel. mRNA levels
Ex6/7 ΔEx6 Ex3/4
f
ZEB1/YFP/DAPI
Fib-WT-ROSA-YFP
Fib-cKO-ROSA-YFP
50μm
g
Ki67/YFP/DAPI
22.3 ± 4.5
23.1 ± 3.8
h
Fib-WT
Fib-cKO
5 weeks
10 weeks
Lactation day 3
4mm
j
P-Fib-WT-ROSA-YFP
P-Fib-cKO-ROSA-YFP
Ki67/YFP/DAPI
Cleaved casp3/YFP/DAPI
l
P-Fib-WT
P-Fib-cKO
n.s.
Ki67+ cells in lung colonies (%)
i
ZEB1/CD31/DAPI
ZEB1/CD45/DAPI
PyMT-Fib-WT
PyMT-Fib-cKO
200μm
k
Ki67

Supplementary Fig. 2 Scheme for the generation of *ZEB1* conditional mice and characterization of FSP1-Cre expression in normal glands and PyMT-induced mammary tumours. a Schematic representation of the *ZEB1* targeted allele. The genomic structure after neo-cassette removal is shown on the third line. The genomic structure after loxP-cassette removal is shown on the fourth line. DTA, diphtheria toxin cassette; red triangle, loxP sites; purple triangle, FRT sites; neo, neomycin resistance cassette. **b** Genotyping assays of tail genomic DNA indicating amplification of the $ZEB1^{wt}$ and $ZEB1^{fl}$ alleles. **c** Genomic DNA analysis for the expression of *ZEB1* exons in $ZEB1^{fl/fl}$ and $ZEB1^{\Delta/\Delta}$ MEFs. MEFs isolated from $ZEB1^{fl/fl}$ embryos are infected with adeno-βGal and -Cre to generate $ZEB1^{fl/fl}$ and $ZEB1^{\Delta/\Delta}$ MEFs and subjected to PCR analysis. Note, exon 6 are selectively deleted in $ZEB1^{\Delta/\Delta}$ MEFs. **d** Positions of specific primers for exon 6/7 (Ex6/7; green), exon 3/4 (Ex3/4; blue) and exon 6-deleted (ΔEx6; red) *ZEB1* transcripts. **e** RT-qPCR analysis of *ZEB1* transcript levels in $ZEB1^{fl/fl}$ and $ZEB1^{\Delta/\Delta}$ MEFs using the indicated primers as described in ***d***. Data are represented as mean ± s.d. (n = 3 independent experiments). ** $P < 0.01$, two-way ANOVA test. **f** Immunofluorescence staining of ZEB1 and GFP in mammary glands of Fib-WT-ROSA-YFP and Fib-cKO-ROSA-YFP reporter mice (the image is representative of images from five mice). Nuclei are counterstained with DAPI. S, stroma; G, gland. **g** Immunofluorescence staining of Ki67 and GFP in mammary glands of Fib-WT-ROSA-YFP and Fib-cKO-ROSA-YFP reporter mice (the image is representative of images from five mice). Nuclei are counterstained with DAPI. S, stroma; G, gland. Data are represented as mean ± s.d. (n = 5 independent experiments). Two-sided Student's t-test. **h** Whole-mount Carmine red staining of mammary glands from Fib-WT and -cKO mice at 5 and 10 weeks of age as well as lactation day 3 (images are representative of images from five mice). **i** Immunofluorescence staining of ZEB1, CD31 (left panels) and CD45 (right panels) in PyMT-Fib-WT and PyMT-Fib-cKO primary tumours (the image is representative of images from five mice). Magnified areas of boxed sections are shown in bottom panels. Arrows denote $ZEB1^{+}/CD31^{+}$ endothelial cells. Nuclei are counterstained with DAPI. **j** Immunofluorescence staining of Ki67 (top panels), cleaved caspase 3 (bottom panels) and GFP in primary tumours of PyMT-Fib-WT-ROSA-YFP and PyMT-Fib-cKO-ROSA-YFP reporter mice (the image is representative of images from five mice). Nuclei are counterstained with DAPI. **k** Immunohistochemical staining of Ki67 in lung nodules of PyMT-Fib-WT and PyMT-Fib-cKO mice (the image is representative of images from six mice). **l** Quantification of Ki67-positive cells in lung nodules as described in ***k***. For quantification, around 1,000 cells are counted in 10 random fields of each section. Data are represented as mean ± s.d. (n = 6 independent experiments). Two-sided Student's t-test. n.s., not significant. The source data are provided as a Source Data file.

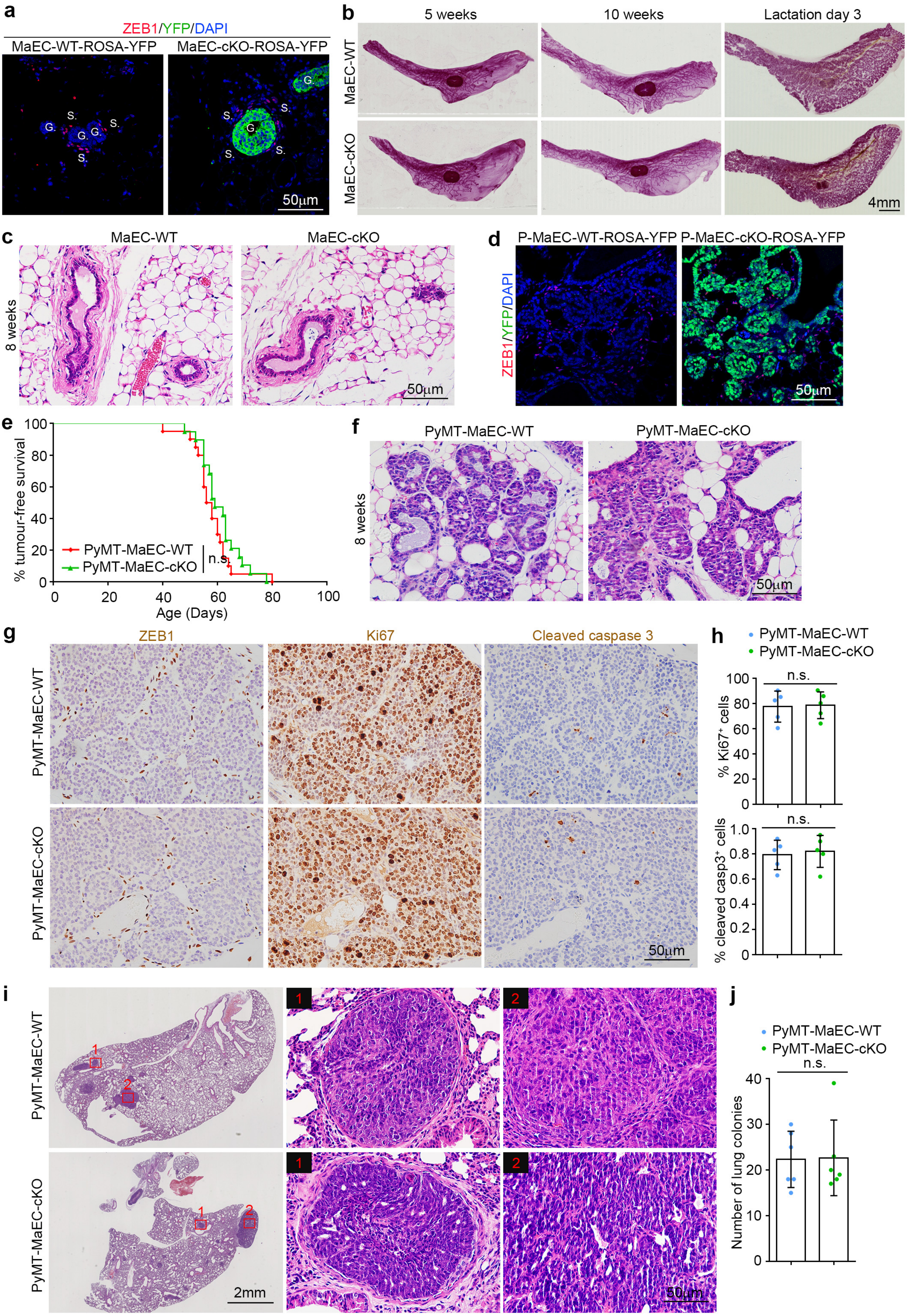

a
ZEB1/YFP/DAPI
MaEC-WT-ROSA-YFP
MaEC-cKO-ROSA-YFP
S.
G.
50μm
b
5 weeks
10 weeks
Lactation day 3
MaEC-WT
MaEC-cKO
4mm
c
MaEC-WT
MaEC-cKO
8 weeks
50μm
d
P-MaEC-WT-ROSA-YFP
P-MaEC-cKO-ROSA-YFP
ZEB1/YFP/DAPI
50μm
e
% tumour-free survival
Age (Days)
PyMT-MaEC-WT
PyMT-MaEC-cKO
n.s.
f
PyMT-MaEC-WT
PyMT-MaEC-cKO
8 weeks
50μm
g
ZEB1
Ki67
Cleaved caspase 3
PyMT-MaEC-WT
PyMT-MaEC-cKO
50μm
h
PyMT-MaEC-WT
PyMT-MaEC-cKO
% Ki67+ cells
% cleaved casp3+ cells
n.s.
i
PyMT-MaEC-WT
PyMT-MaEC-cKO
2mm
50μm
j
PyMT-MaEC-WT
PyMT-MaEC-cKO
n.s.
Number of lung colonies

Supplementary Fig. 3 ZEB1 deletion in MaECs does not affect normal gland development or PyMT-induced tumour progression. a Immunofluorescence staining of ZEB1 and GFP in mammary glands of MaEC-WT-ROSA-YFP and -cKO-ROSA-YFP reporter mice (the image is representative of images from five mice). Nuclei are counterstained with DAPI. **b** Whole-mount Carmine red staining of mammary glands from MaEC-WT and -cKO mice at 5 and 10 weeks of age as well as lactation day 3 (images are representative of images from five mice). **c** H.E. staining of mammary glands from MaEC-WT and -cKO mice at 8 weeks of age (images are representative of images from five mice). **d** Immunofluorescence staining of ZEB1 and GFP in primary tumours of PyMT-MaEC-WT-ROSA-YFP and -cKO-ROSA-YFP reporter mice (the image is representative of images from five mice). Nuclei are counterstained with DAPI. **e** Kaplan-Meier analysis of mammary tumour progression in PyMT-MaEC-WT (n = 20 mice) and -cKO (n = 19 mice) females. n.s., not significant; log-rank test. **f** H.E. staining of primary tumours from PyMT-MaEC-WT and -cKO mice at 8 weeks of age (images are representative of images from five mice). **g** Immunohistochemical staining of ZEB1 (left panels), Ki67 (middle panels) and cleaved caspase 3 (right panels) in PyMT-MaEC-WT and -cKO mice (the image is representative of images from five mice). **h** Quantification of Ki67- and cleaved caspase 3-positive cells in the indicated primary tumours as described in ***g***. For quantification, around 1,000 cells are counted in 10 random fields of each section. Data are represented as mean ± s.d. (n = 5 independent experiments). Two-sided Student's t-test. ns, not significant. **i** H.E. staining of lungs from PyMT-MaEC-WT and -cKO mice at 9 weeks post-detection of mammary tumours (n = 6 mice, each). Magnified areas of boxed sections are shown in right panels. **j** Number of lung colonies in the cohorts as shown in ***i***. Two-sided Student's t-test. n.s., not significant. The source data are provided as a Source Data file.

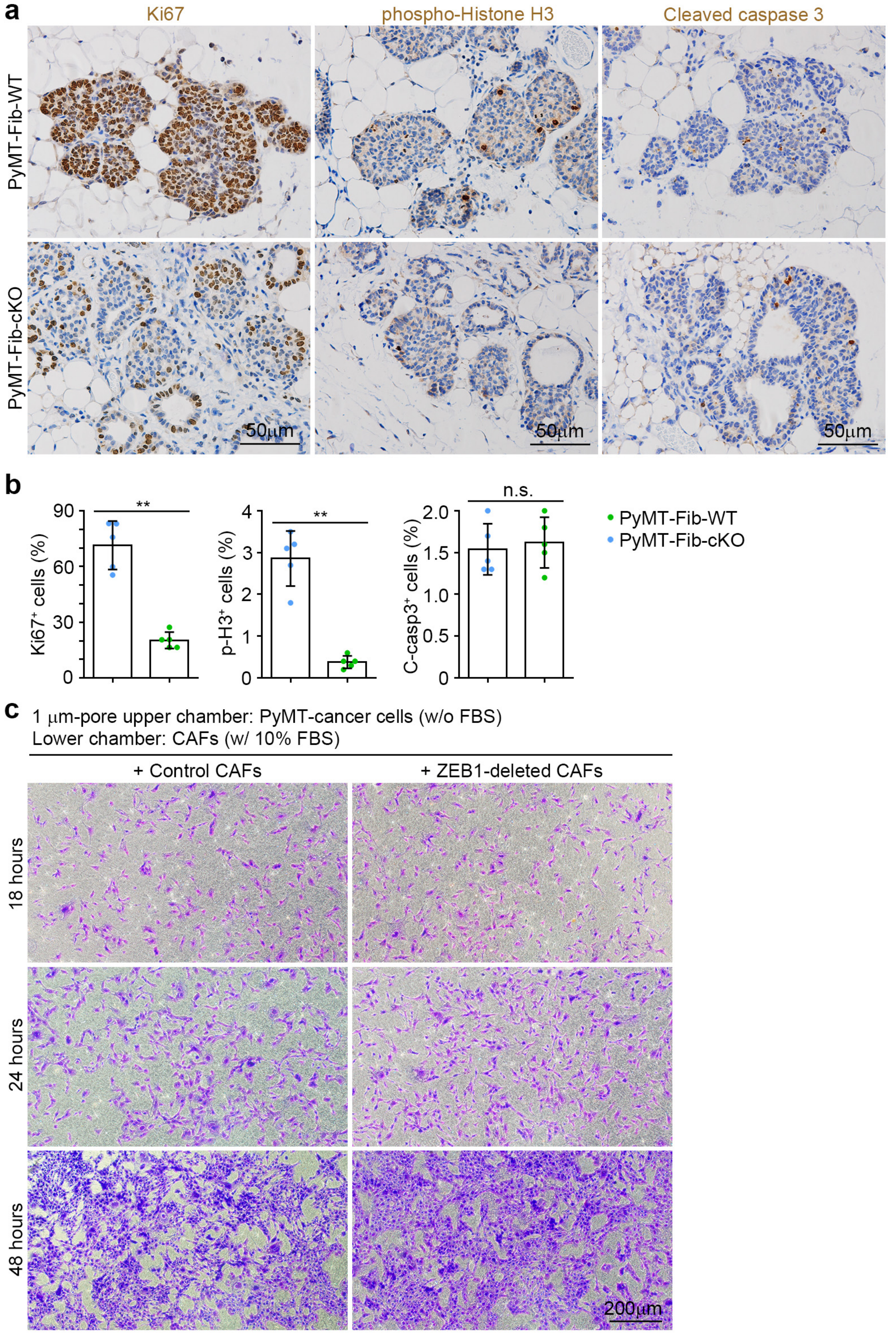
a
Ki67
phospho-Histone H3
Cleaved caspase 3
PyMT-Fib-WT
PyMT-Fib-cKO
50μm
50μm
50μm
b
Ki67+ cells (%)
90
60
30
0
**
p-H3+ cells (%)
4
3
2
1
0
**
C-casp3+ cells (%)
2.0
1.5
1.0
0.5
0
n.s.
PyMT-Fib-WT
PyMT-Fib-cKO
c
1 μm-pore upper chamber: PyMT-cancer cells (w/o FBS)
Lower chamber: CAFs (w/ 10% FBS)
+ Control CAFs
+ ZEB1-deleted CAFs
18 hours
24 hours
48 hours
200μm

Supplementary Fig. 4 *ZEB1* deletion in stromal fibroblasts reduces mammary tumour growth and collective invasion. a Immunohistochemical analyses of Ki67, phospho-histone H3 and cleaved caspase 3 in early-stage primary tumours derived from PyMT-Fib-WT and -cKO mice (images are representative of images from five mice). The arrows in the bottom panels denote cells positive for cleaved caspase 3 (*i.e.* apoptotic cells). **b** Quantification of Ki67-, phospho-histone H3 (p-H3)- and cleaved caspase 3 (C-casp3)-positive cells in the indicated primary tumours as described in ***a***. For quantification, around 1,000 cells are counted in 10 random fields of each section. Data are represented as mean ± s.d. (n = 5 independent experiments). ** $P < 0.01$, two-sided Student's t-test. n.s., not significant. **c** Boyden chamber invasion assay of PyMT-cancer cells. 4×10^4 stromal CAFs were seeded in medium with 10% FBS in the lower chambers and allowed to attach for 24 h, and then 1×10^5 cancer cells were placed in medium without FBS in the upper chambers of transwell inserts with a 1 μm-pore size pre-coated with diluted Matrigel. Under these conditions, cells can proliferate but cannot invade into the bottom chamber. Cells were stained with crystal violet and counted (representative images are from three independent experiments). The source data are provided as a Source Data file.

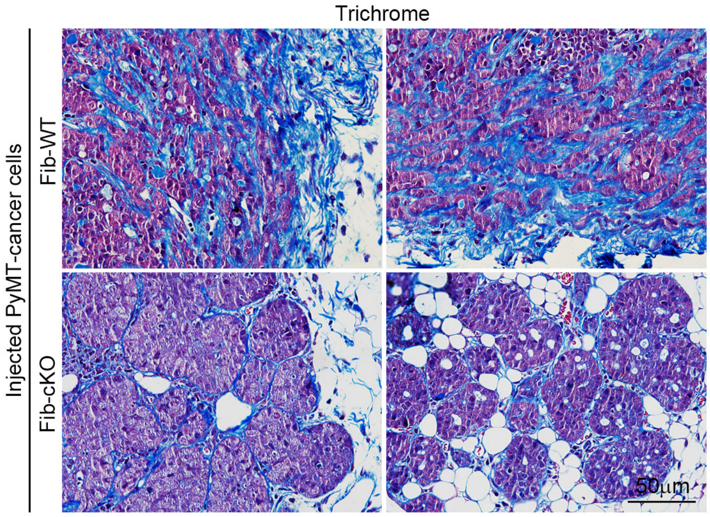
Trichrome
Injected PyMT-cancer cells
Fib-WT
Fib-cKO
50µm

Supplementary Fig. 5 *ZEB1* deletion in stromal fibroblasts suppresses collagen deposition in orthotopic tumours formed in Fib-WT and -cKO mammary glands. Masson's trichrome staining of the orthotopic tumours (edge areas) from the indicated mice (images are representative of images from five mice).

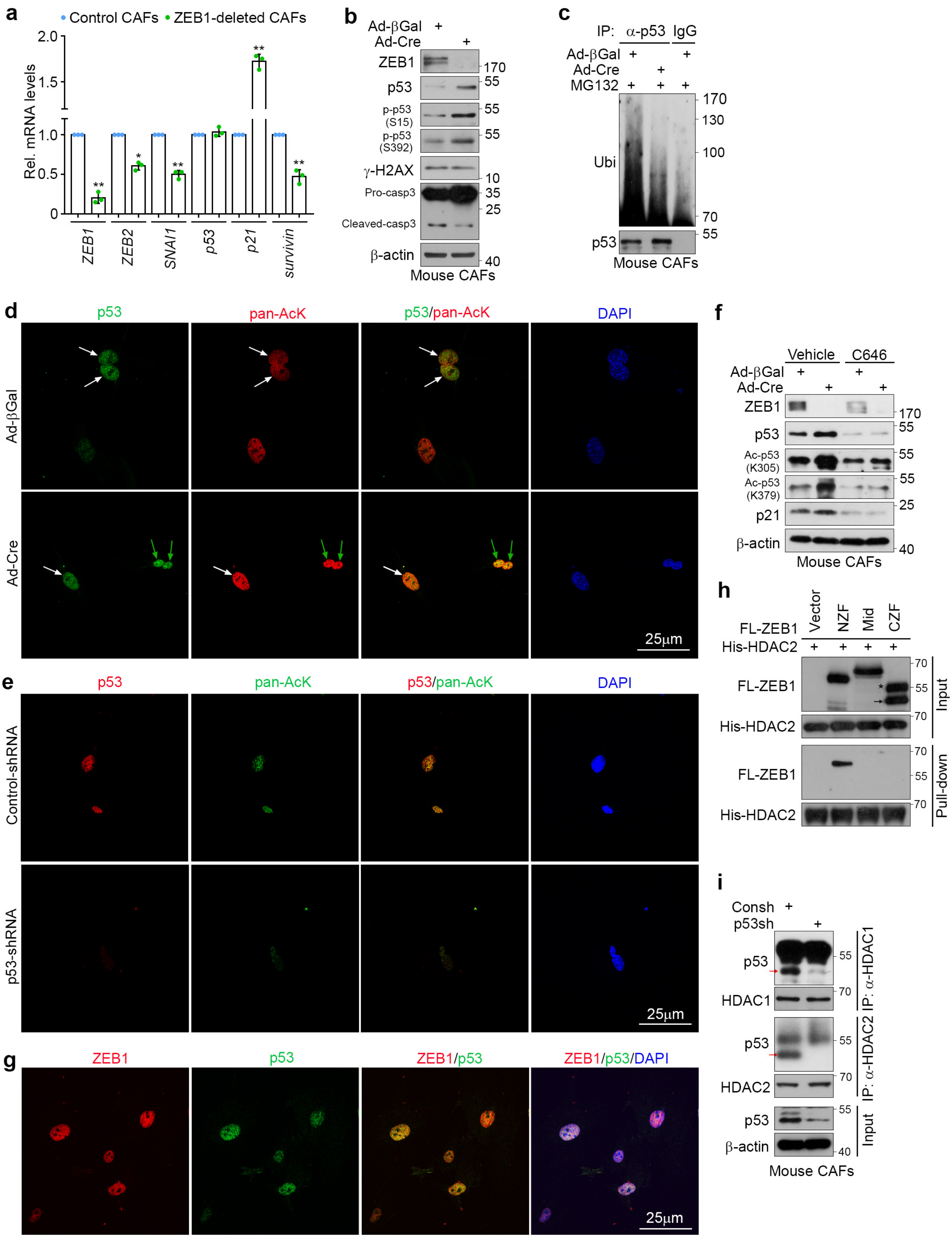

a
Control CAFs
ZEB1-deleted CAFs
Rel. mRNA levels
2.0
1.5
1.0
0.5
0
**
*
**
**
**
ZEB1
ZEB2
SNAI1
p53
p21
survivin
b
Ad-βGal
Ad-Cre
ZEB1
p53
p-p53 (S15)
p-p53 (S392)
γ-H2AX
Pro-casp3
Cleaved-casp3
β-actin
170
55
10
35
25
40
Mouse CAFs
c
IP: α-p53 IgG
Ad-βGal
Ad-Cre
MG132
Ubi
p53
170
130
100
70
55
Mouse CAFs
d
p53
pan-AcK
p53/pan-AcK
DAPI
Ad-βGal
Ad-Cre
25μm
e
p53
pan-AcK
p53/pan-AcK
DAPI
Control-shRNA
p53-shRNA
25μm
f
Vehicle
C646
Ad-βGal
Ad-Cre
ZEB1
p53
Ac-p53 (K305)
Ac-p53 (K379)
p21
β-actin
170
55
25
40
Mouse CAFs
g
ZEB1
p53
ZEB1/p53
ZEB1/p53/DAPI
25μm
h
FL-ZEB1
Vector
NZF
Mid
CZF
His-HDAC2
FL-ZEB1
His-HDAC2
70
55
Input
Pull-down
i
Consh
p53sh
p53
HDAC1
p53
HDAC2
p53
β-actin
IP: α-HDAC1
IP: α-HDAC2
Input
55
70
40
Mouse CAFs

Supplementary Fig. 6 Loss of ZEB1 upregulates p53 protein by increasing its acetylation levels in stromal CAFs. a RT-qPCR analyses for *ZEB1*, *ZEB2*, *SNAI1*, *p53*, *p21* and *survivin* in primarily cultured control and ZEB1-deleted CAFs. Data are represented as mean ± s.d. (n = 3 independent experiments). * $P < 0.05$, ** $P < 0.01$, two-sided Student's t-test. **b** Immunoblot analysis of primarily cultured control and ZEB1-deleted CAFs using the indicated antibodies. **c** Lysates from MG132-treated (10 μM for 6 h) control and ZEB1-deleted CAFs were subjected to immunoprecipitation (IP) assay. **d** Immunofluorescence staining of p53 and pan-AcK in primarily cultured control and ZEB1-deleted CAFs (representative images are from three independent experiments). Nuclei are DAPI-stained. White arrows denote the cells expressing comparable levels of p53 between control and ZEB1-deleted cells, whereas green arrows mark the cells expressing higher levels of p53 in control group as compared to ZEB1-deleted group. **e** Immunofluorescence staining of p53 and pan-AcK in primarily cultured control-shRNA and p53-shRNA infected CAFs (representative images are from three independent experiments). Nuclei are DAPI-stained. **f** Control and ZEB1-deleted CAFs were treated with C646 (20 μM for 48 h), and cell lysates were collected for immunoblot assays using the indicated antibodies. **g** Immunofluorescence staining of ZEB1 and p53 in primarily cultured control CAFs (representative images are from three independent experiments). Nuclei are DAPI-stained. **h** Recombinant His-HDAC2 protein bound to Ni-NTA beads was co-incubated with FLAG-ZEB1 fragments, and the mixture was subjected to His pull-down assays. **i** Lysates from Control-shRNA and p53-shRNA infected CAFs were subjected to co-IP assays using anti-HDAC1/2 antibodies. Asterisks and arrows mark non-specific bands and specific bands with the expected molecular weights, respectively. All representative blots shown are from three independent experiments. Unprocessed original scans of blots are shown in Supplementary Fig. 9. The source data are provided as a Source Data file.

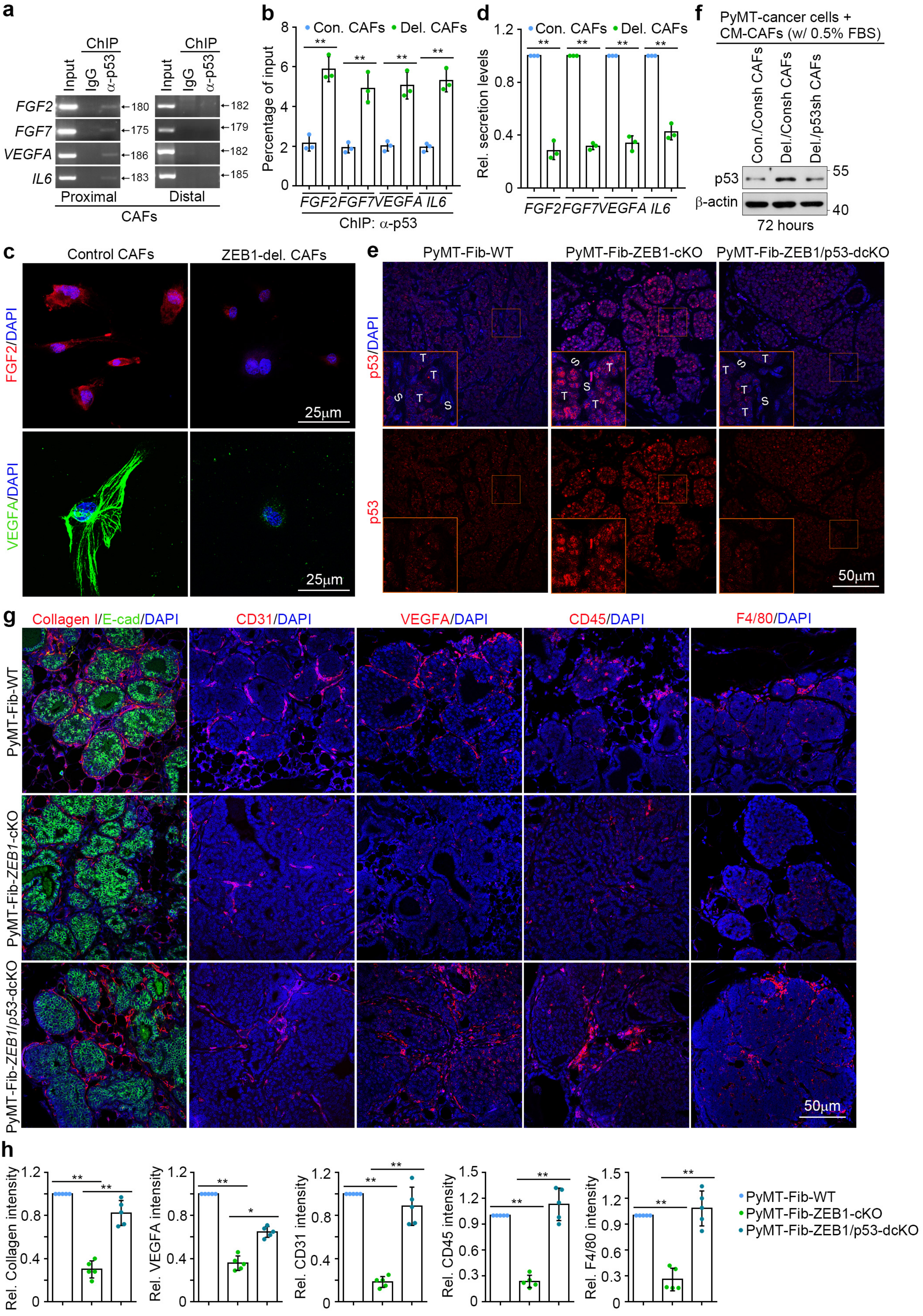

a
ChIP
Input
IgG
α-p53
FGF2
FGF7
VEGFA
IL6
←180
←175
←186
←183
←182
←179
←182
←185
Proximal
Distal
CAFs
b
Con. CAFs
Del. CAFs
Percentage of input
FGF2 FGF7 VEGFA IL6
ChIP: α-p53
d
Rel. secretion levels
f
PyMT-cancer cells +
CM-CAFs (w/ 0.5% FBS)
Con./Consh CAFs
Del./Consh CAFs
Del./p53sh CAFs
p53
β-actin
55
40
72 hours
c
Control CAFs
ZEB1-del. CAFs
FGF2/DAPI
VEGFA/DAPI
25μm
e
PyMT-Fib-WT
PyMT-Fib-ZEB1-cKO
PyMT-Fib-ZEB1/p53-dcKO
p53/DAPI
p53
50μm
g
Collagen I/E-cad/DAPI
CD31/DAPI
VEGFA/DAPI
CD45/DAPI
F4/80/DAPI
PyMT-Fib-WT
PyMT-Fib-ZEB1-cKO
PyMT-Fib-ZEB1/p53-dcKO
50μm
h
Rel. Collagen intensity
Rel. VEGFA intensity
Rel. CD31 intensity
Rel. CD45 intensity
Rel. F4/80 intensity
PyMT-Fib-WT
PyMT-Fib-ZEB1-cKO
PyMT-Fib-ZEB1/p53-dcKO

Supplementary Fig. 7 A stromal ZEB1/p53 axis regulates mammary tumour growth and progression in a paracrine fashion. a Chromatin immunoprecipitation (ChIP)/PCR analyses for the interactions between p53 and proximal (left panels) and distal (distal) promoters of *FGF2*, *FGF7*, *VEGFA* or *IL6* genes in stromal CAFs. All representative blots shown are from three independent experiments. **b** Quantification of the interactions between p53 and *FGF2*, *FGF7*, *VEGFA* or *IL6* promoters in control and ZEB1-deleted CAFs as assessed by ChIP/qPCR analysis. Data are represented as mean ± s.d. (n = 3 independent experiments). ** $P < 0.01$, two-sided Student's t-test. **c** Immunofluorescence staining of FGF2 (left panels) and VEGFA (right panels) in primarily cultured control and ZEB1-deleted CAFs (representative images are from three independent experiments). Nuclei are DAPI-stained. **d** ELISA analysis of the indicated secreted proteins in control and ZEB1-deleted CAFs. Data are represented as mean ± s.d. (n = 3 independent experiments). ** $P < 0.01$, two-sided Student's t-test. **e** Immunofluorescence staining of p53 in primary tumours derived from PyMT-Fib-WT, -ZEB1-cKO and -ZEB1/p53-dcKO females (images are representative of images from five mice). Insets display higher magnifications of boxed areas. **f** PyMT-cancer cells were incubated for 72 h in the presence of conditioned medium (containing 0.5% FBS) generated from the indicated CAFs, and cell lysates were collected for immunoblot assays. Representative blots shown are from three independent experiments. **g** Immunofluorescence staining of type I collagen, CD31, VEGFA, CD45 and F4/80 in primary tumours derived from PyMT-Fib-WT, -ZEB1-cKO and -ZEB1/p53-dcKO females (images are representative of images from five mice). **h** Quantification of relative fluorescent intensities in 10 random fields of each section of the indicated primary tumours as described in ***g***. Data are represented as mean ± s.d. (n = 5 independent experiments). * $P < 0.05$, ** $P < 0.01$, two-sided Student's t-test. Unprocessed original scans of blots are shown in Supplementary Fig. 9. The source data are provided as a Source Data file.

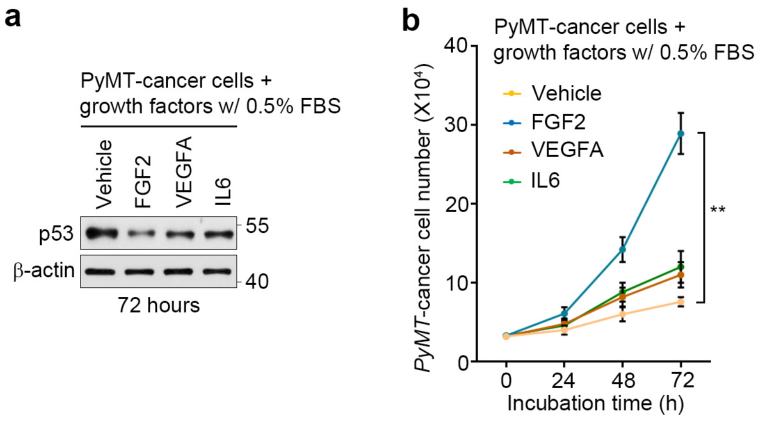
a
PyMT-cancer cells +
growth factors w/ 0.5% FBS
Vehicle
FGF2
VEGFA
IL6
p53
β-actin
55
40
72 hours
b
PyMT-cancer cells +
growth factors w/ 0.5% FBS
Vehicle
FGF2
VEGFA
IL6
**
PyMT-cancer cell number (X10⁴)
0
10
20
30
40
0
24
48
72
Incubation time (h)

Supplementary Fig. 8 FGF2 decreases p53 protein levels while promoting proliferation in PyMT-cancer cells. a PyMT-cancer cells were incubated for 72 h in the presence of culture medium supplemented with 0.5% FBS and FGF2 (20 ng/ml), VEGFA (20 ng/ml) or IL6 (100 ng/ml), and cell lysates were collected for immunoblot assays. Representative blots shown are from three independent experiments. **b** 3×10^4 PyMT-cancer cells were cultured in medium with 10% FBS overnight, and cells were extensively washed with PBS and refed with fresh medium supplemented with 0.5% FBS and FGF2 (20 ng/ml), VEGFA (20 ng/ml) or IL6 (100 ng/ml). Cell numbers were counted under a microscope at 24, 48 and 72 hours of culture. Data are represented as mean ± s.d. (n = 3 independent experiments). ** $P < 0.01$, two-way ANOVA test. Unprocessed original scans of blots are shown in Supplementary Fig. 9. The source data are provided as a Source Data file.

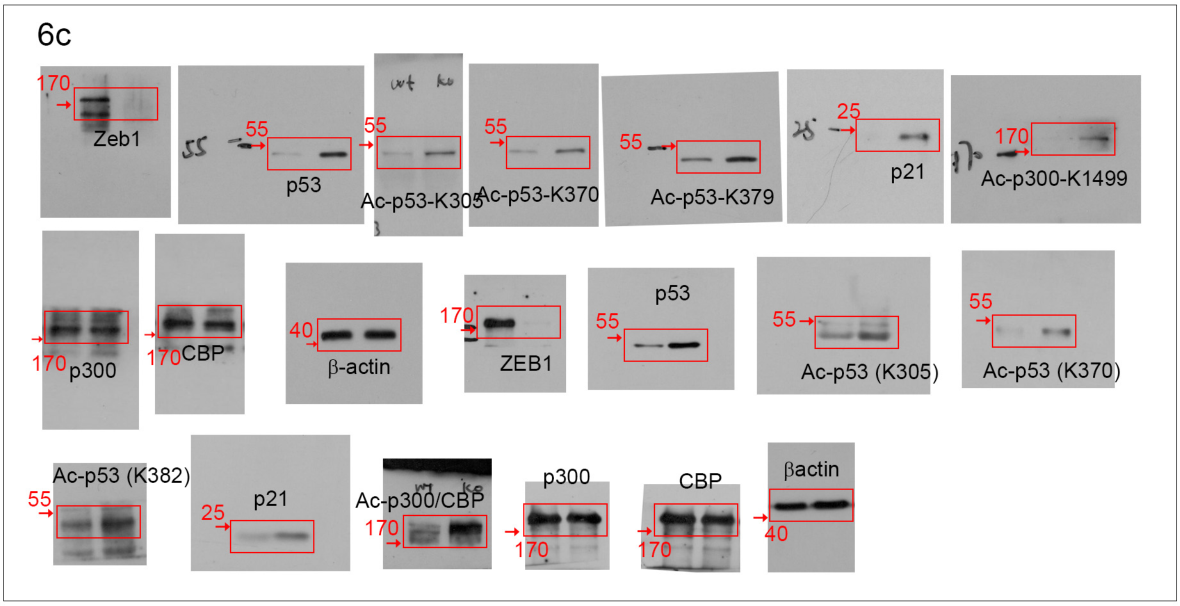
6c
170
Zeb1
55
p53
55
wt ko
Ac-p53-K305
55
Ac-p53-K370
55
Ac-p53-K379
25
p21
170
Ac-p300-K1499
170
p300
170
CBP
40
β-actin
170
ZEB1
p53
55
55
Ac-p53 (K305)
55
Ac-p53 (K370)
Ac-p53 (K382)
55
p21
25
Ac-p300/CBP
170
p300
170
CBP
170
βactin
40

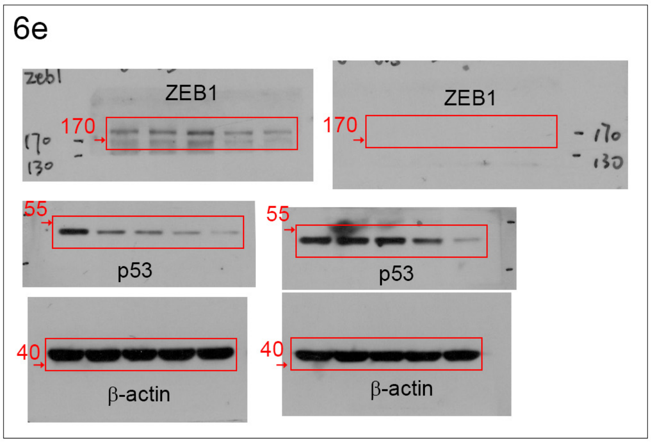
6e
ZEB1
170
ZEB1
170
55
p53
55
p53
40
β-actin
40
β-actin

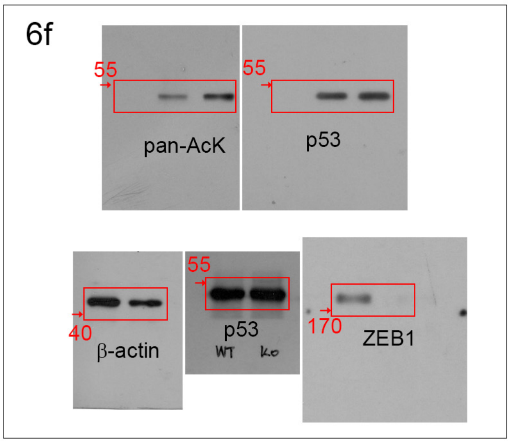
6f
55
pan-AcK
55
p53
40
β-actin
55
p53
WT ko
170
ZEB1

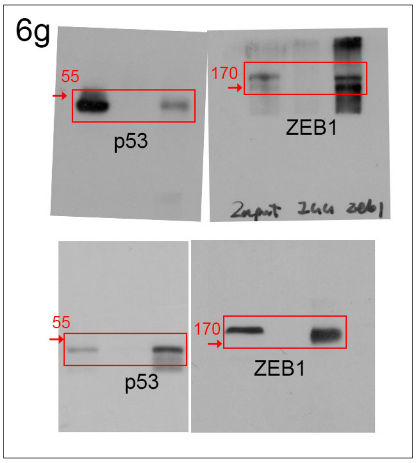
6g
55
p53
170
ZEB1
55
p53
170
ZEB1

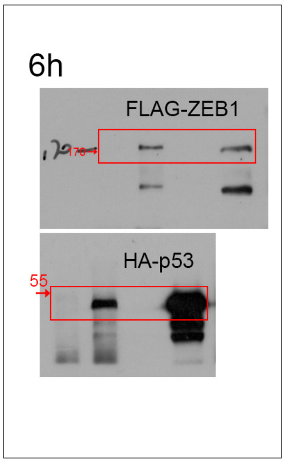
6h
FLAG-ZEB1
HA-p53
55

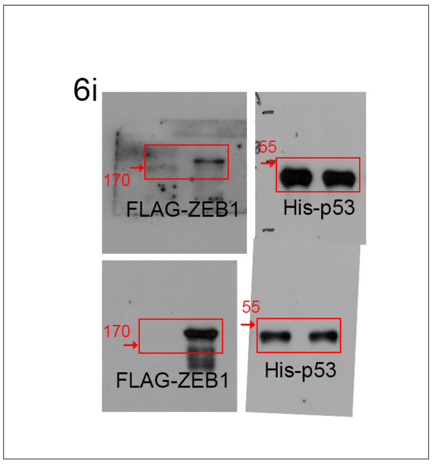
6i
170
FLAG-ZEB1
55
His-p53
170
FLAG-ZEB1
55
His-p53

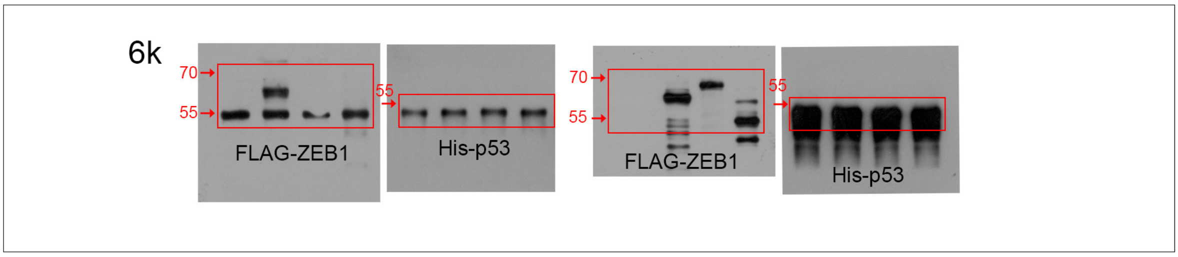
6k
70
55
FLAG-ZEB1
55
His-p53
70
55
FLAG-ZEB1
55
His-p53

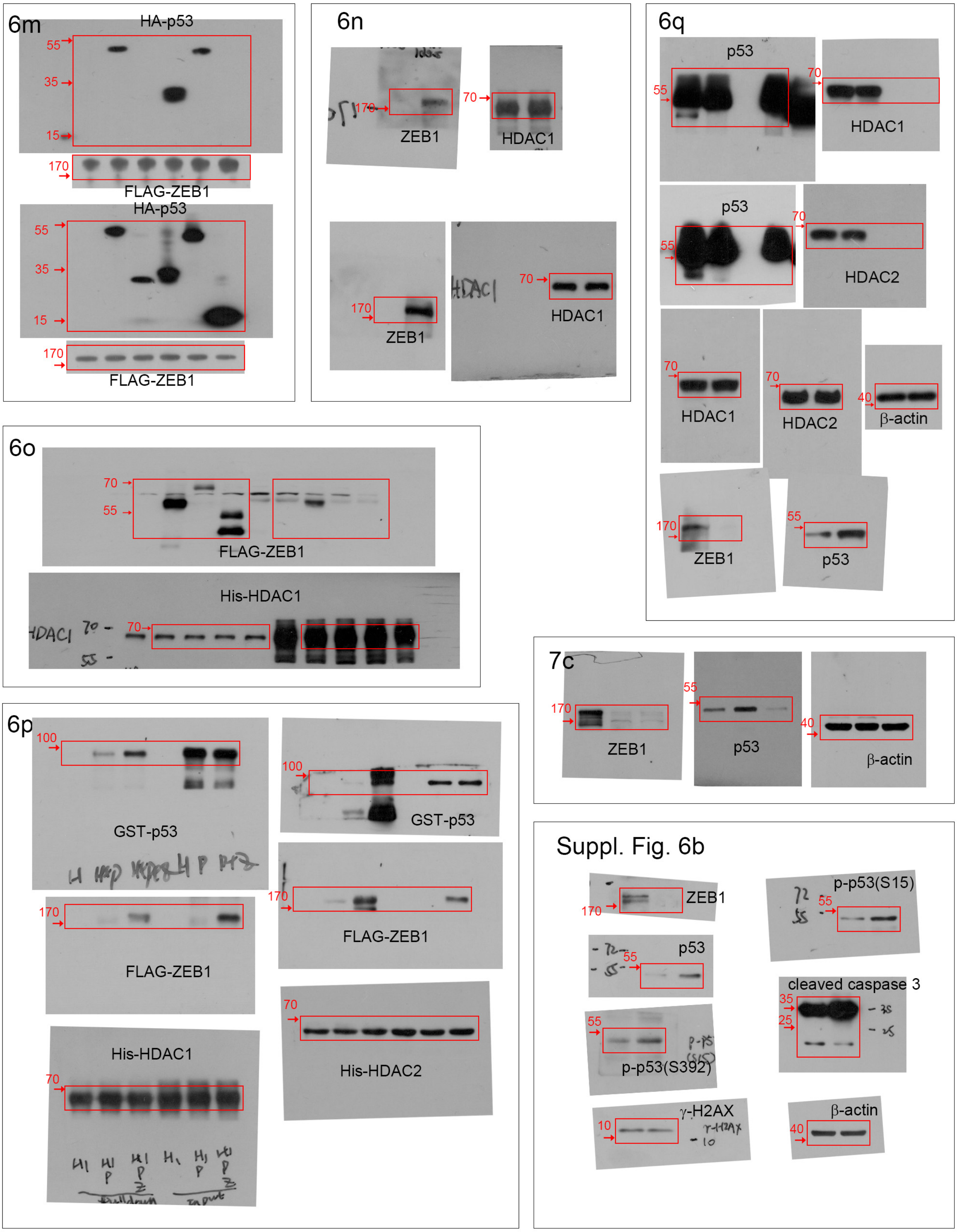
6m
HA-p53
55
35
15
170
FLAG-ZEB1
HA-p53
55
35
15
170
FLAG-ZEB1
6n
170
ZEB1
70
HDAC1
170
ZEB1
70
HDAC1
6q
p53
55
70
HDAC1
p53
55
70
HDAC2
70
HDAC1
70
HDAC2
40
β-actin
170
ZEB1
55
p53
6o
70
55
FLAG-ZEB1
His-HDAC1
70
6p
100
GST-p53
100
GST-p53
170
FLAG-ZEB1
170
FLAG-ZEB1
His-HDAC1
70
70
His-HDAC2
7c
170
ZEB1
55
p53
40
β-actin
Suppl. Fig. 6b
170
ZEB1
p-p53(S15)
55
p53
55
cleaved caspase 3
35
25
55
p-p53(S392)
γ-H2AX
10
β-actin
40

Suppl. 6c

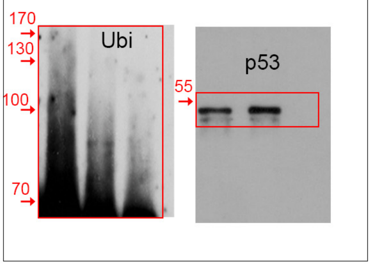

Suppl. 6f

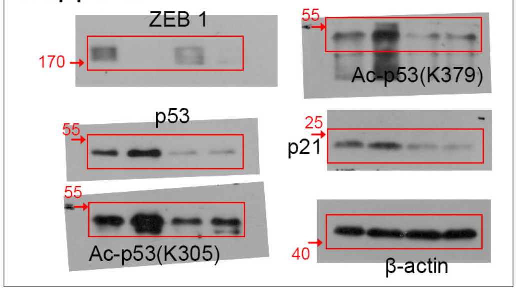

Suppl. 6h

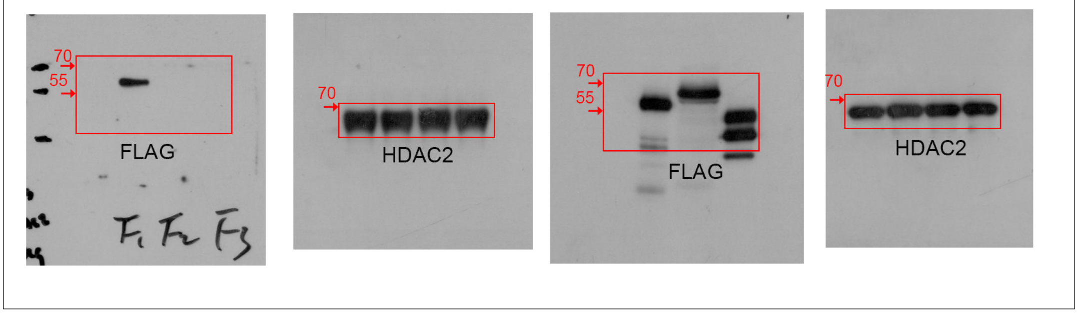

Suppl. 6i

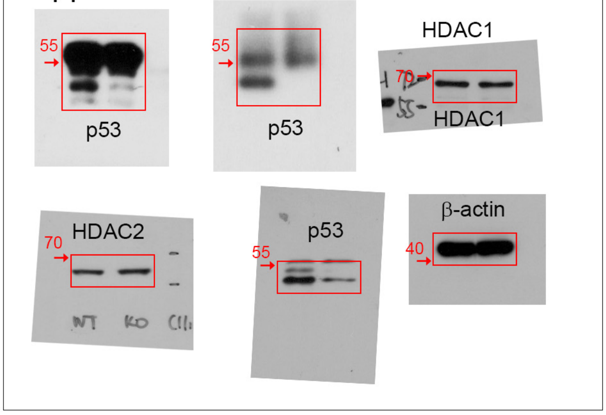

Suppl. 7f

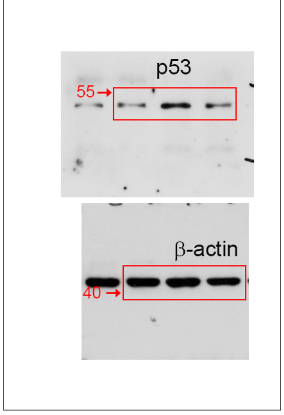

Suppl. 8a

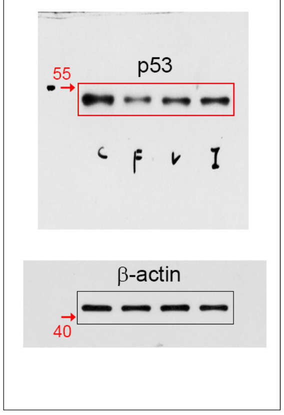

Supplementary Fig. 9 Unprocessed images of blots. Uncropped images of scanned immunoblots shown in figures and supplementary figures are provided.

Supplementary Table 1.

Primer sets for genotyping

Primer name	Sequence (5'-3')	
ZEB1-flox/wt (flox, 296 bp; wt, 197 bp)	Forward	GGTTTTACCATGCCCACTAATATGA
	Reverse	GAGGCAAGAAAAACAAATGTAATCTCC
MMTV-PyMT (PCR product, 556 bp)	Forward	GGAAGCAAGTACTTCACAAGGG
	Reverse	GGAAAGTCACTAGGAGCAGGG
Fsp1-Cre (PCR product, 1082 bp)	Forward	ATGCTTCTGTCCGTTTGCCG
	Reverse	CAATGCGATGCAATTTCCTC
MMTV-Cre (PCR product, 280 bp)	Forward	GGTTCTGATCTGAGCTCTGAGTG
	Reverse	CATCACTCGTTGGATCGACCGG
p53-flox/wt (flox, 390 bp; wt, 270 bp)	Forward	GGTTAAACCCAGCTTGACCA
	Reverse	GGAGGCAGAGACAGTTGGAG
MMTV-ErbB2/neu (PCR product, 79 bp)	Forward	CCCCGGGAGTATGTGAGTGA
	Reverse	TGAGCTGTTTTGAGGCTGACA
MMTV-Wnt1 (PCR product, 440 bp)	Forward	GGACTTGCTTCTCTTCTCATAGCC
	Reverse	CCACACAGGCATAGAGTGTCTGC

Primer sets for qPCR or PCR

**Primer name**	**Forward primer**	**Reversed primer**
Krt8	GCTGAAGCTGGAGGCTGAGCT	AGGCGGGACTCTAGTTCCACC
Cdh2	TGTGCACGAAGGACAGCCCCT	CCTGCTCTGCAGTGAGAGGGA
Cdh1	GAAGTCCATGGGGCACCACCA	CTGAGACCTGGGTACACGCTG
Fn1	GGGTGACACTTATGAGCGCCC	GACTGACCCCCTTCATGGCAG
PyMT	GCCTATAGACTGGCTGGACC	CTCTCCTCAGTTCCTCGCTCC
Zeb2	GCACCCAGCTCGAGAGGCATA	AAGGCCTTGCCACACTCCGTG
Snai1	ATAGCGAGCTGCAGGACGCGT	AGATGAGGGTGGGCAGCGAAG
p53	AGCTCCCTCTGAGCCAGGAGA	TCCTCAACATCCTGGGGCAGC
p21	TGCCGTTGTCTCTTCGGTCCC	TAGACCTTGGGCAGCCCTAGG
p16	AGCTGCGCTCTGGCTTTCGTG	GCTGCTACGTGAACGTTGCCC
Fgf2	AGAACGGCGGCTTCTTCCTGC	CCAGCAGCCGTCCATCTTCCT
Fgf7	CCGTGGCAGTTGGAATTGTGGC	CCCCTCCGCTGTGTGTCCATT
Fgf10	CTGTCCGTACAGTGTCCTGGAG	GCCTGCCATTGTGCTGCCAGT
Vegfa	GCACATAGAGAGAATGAGCTTCC	CTCCGCTCTGAACAAGGCT
IL6	GCCAGAGTCCTTCAGAGAGAT	ACTCCTTCTGTGACTCCAGCT
Hgf	AGCAGACACCACACCGGCACA	CCAAGGGGTGTCAGGGTCAAG
Tgfb1	ACATGAACCGGCCCTTCCTGC	TACAGCTGCCGCACACAGCAG
Tgfb2	AACCCCAAAGCCAGAGTGGCC	TCCTGCACAGCGTCTGTCACG
Tgfb3	AACCCCAGCTCCAAGCGCACA	ATTCAGCGGTGCCCCTTGTGG
Igf1	GGCTGAGCTGGTGGATGCTCT	CTCCTCAGATCACAGCTCCGG
Igf2	AGAGACTCTGTGCGGAGGGGA	CAGCTGCGGAAGCAGCACTCT
Egf	AGTACACCCTCGTGCACAGCC	TCGAGTGGGACTTGGGGTCTC
Gapdh	CCCTGGCCAAGGTCATCCATG	TGATGTTCTGGGCAGCCCCAC
Zeb1-exon 3/4	TTATCCTGAGGCGCCCGAGGA	TACGGGCAGGTGAGCAACTGG
Zeb1-exon 6/7	CTGGCCGCCAACAAGCAGACT	CCTCCACAGTGGAGACTCCTTC
Zeb1-Δ exon 6	GCACTGAATGCGGGAAGGCCT	CCTCCACAGTGGAGACTCCTTC
Zeb1-exon 1	TCAAACTCTGCAGCGTCCAAG	ACATAACGGTCCAGTCCAGCA
Zeb1-exon 2	GTCACTGTTGACCATCTGGATC	CGTTGTCTTGCCAGCAGTTCT
Zeb1-exon 3	GCACAGCTCTGAAGCATAAGG	CATCATGACTGCTGGCTTCTG
Zeb1-exon 4	CTCATGCATACCTGGAGGTGT	GCTCTCTTCCTGACTTATGTGA
Zeb1-exon 5	TAAGACAAAGGCCGAACAGCC	CCACTGTGAATCCGTAAGTGC
Zeb1-exon 6	AGCTTGATGCCTGTGAATGGC	GCGAAGCACTCACTGCTTCTT
Zeb1-exon 7	GACACTAGCTCAGAAGGAGTC	TGTGAAAAGTGGAGACCACGG
Zeb1-exon 8	TTGATTGAGCACATGCGGCTG	CTTCTCTTCAGACAGCTGCTC

Primer sets for Chromatin immunoprecipitation (ChIP)

Primer name	Forward primer	Reversed primer
FGF2 (PCR product, 180 bp)	AGAAAGCTCTGCCTAGCGGGA	TGCAGCCGGACTCTTCCTGAA
FGF7 (PCR product, 175 bp)	CTCCTCCGTGCCAATGTATTGT	GCATTCACGCTGTGGAAACAGA
VEGFA (PCR product, 186 bp)	TGAGGCCGTGGACCCTGGTAA	AAGCCTCTGCGCTTCTCACCG
IL6 *(PCR product, 183 bp)*	GACGTCACATTGTGCAATCTT	GGGCTCCAGAGCAGAATGAGC
