# Supplementary material for: A ZEB1/p53 signaling axis in stromal fibroblasts promotes mammary epithelial tumours
Source: Nat Commun. 2019 Jul 19;10:3210. doi: 10.1038/s41467-019-11278-7 (PMC6642263; doi:10.1038/s41467-019-11278-7)
Supplement: Supplementary file 2 — Reporting Summary [file 41467_2019_11278_MOESM2_ESM.pdf]

nature research

Corresponding author(s): Zhao-Qiu Wu

Last updated by author(s): June 15, 2019

Reporting Summary

Nature Research wishes to improve the reproducibility of the work that we publish. This form provides structure for consistency and transparency in reporting. For further information on Nature Research policies, see Authors & Referees and the Editorial Policy Checklist.

Statistics

For all statistical analyses, confirm that the following items are present in the figure legend, table legend, main text, or Methods section.

n/a	Confirmed	
☐	☒	The exact sample size (*n*) for each experimental group/condition, given as a discrete number and unit of measurement
☐	☒	A statement on whether measurements were taken from distinct samples or whether the same sample was measured repeatedly
☐	☒	The statistical test(s) used AND whether they are one- or two-sided<br>*Only common tests should be described solely by name; describe more complex techniques in the Methods section.*
☒	☐	A description of all covariates tested
☒	☐	A description of any assumptions or corrections, such as tests of normality and adjustment for multiple comparisons
☐	☒	A full description of the statistical parameters including central tendency (e.g. means) or other basic estimates (e.g. regression coefficient) AND variation (e.g. standard deviation) or associated estimates of uncertainty (e.g. confidence intervals)
☐	☒	For null hypothesis testing, the test statistic (e.g. *F*, *t*, *r*) with confidence intervals, effect sizes, degrees of freedom and *P* value noted<br>*Give P values as exact values whenever suitable.*
☒	☐	For Bayesian analysis, information on the choice of priors and Markov chain Monte Carlo settings
☒	☐	For hierarchical and complex designs, identification of the appropriate level for tests and full reporting of outcomes
☐	☒	Estimates of effect sizes (e.g. Cohen's *d*, Pearson's *r*), indicating how they were calculated

Our web collection on statistics for biologists contains articles on many of the points above.

Software and code

Policy information about availability of computer code

Data collection	Immunofluorescent stainings were analysed with Image J software.<br>For flow cytometry, data were collected on a BD FACS Verse flow cytometer.<br>Q-PCR was performed using an ABI 7300 sequence detection system.
Data analysis	GraphPad Prism software.

For manuscripts utilizing custom algorithms or software that are central to the research but not yet described in published literature, software must be made available to editors/reviewers. We strongly encourage code deposition in a community repository (e.g. GitHub). See the Nature Research guidelines for submitting code & software for further information.

Data

Policy information about availability of data

All manuscripts must include a data availability statement. This statement should provide the following information, where applicable:

- Accession codes, unique identifiers, or web links for publicly available datasets
- A list of figures that have associated raw data
- A description of any restrictions on data availability

Patient data supporting the analyses in Figs. 1c-g, 7b and Supplementary Fig. 1 are accessed from the NCBI gene expression omnibus (GSE9014), TCGA RNA-Seq gene expression profiles data for breast cancer (https://portal.gdc.cancer.gov/) and the METABRIC microarray gene expression profiles data for breast cancer (http://www.cbioportal.org). The source data underlying Figs. 1b-g, 1j, 2d, 2e, 2g, 2i, 2j, 3c, 3e, 4b, 4e, 4f, 4h, 5a, 5d, 5f, 6b, 6e, 6r, 7a, 7b, 7d, 7e, 7g, 7i, 7k and Supplementary Figs. 2e, 2l, 3e, 3h, 3j, 4b, 6a, 7b, 7d, 7h, 8b are provided as a Source Data file. Unprocessed original scans of blots are shown in Supplementary Fig. 9. All data supporting the findings of this study are available from the corresponding author on reasonable request. A reporting summary for this Article is available as a Supplementary Information file.

Field-specific reporting

Please select the one below that is the best fit for your research. If you are not sure, read the appropriate sections before making your selection.

☒ Life sciences ☐ Behavioural & social sciences ☐ Ecological, evolutionary & environmental sciences

For a reference copy of the document with all sections, see nature.com/documents/nr-reporting-summary-flat.pdf

Life sciences study design

All studies must disclose on these points even when the disclosure is negative.

Sample size	There were at least 3 samples (mice) in independent experiments.
Data exclusions	No animals or samples were excluded from analysis.
Replication	Experiments were repeated at least 3 times to ensure reproducibility except where otherwise indicated in the figure legends.
Randomization	No method of randomization was used as mice were segregated into groups based on genotype alone.
Blinding	The investigators were not blinded to allocation during experiments and outcome assessment.

Reporting for specific materials, systems and methods

We require information from authors about some types of materials, experimental systems and methods used in many studies. Here, indicate whether each material, system or method listed is relevant to your study. If you are not sure if a list item applies to your research, read the appropriate section before selecting a response.

Materials & experimental systems

n/a	Involved in the study	
☐	☒	Antibodies
☐	☒	Eukaryotic cell lines
☒	☐	Palaeontology
☐	☒	Animals and other organisms
☒	☐	Human research participants
☒	☐	Clinical data

Methods

n/a	Involved in the study	
☒	☐	ChIP-seq
☐	☒	Flow cytometry
☒	☐	MRI-based neuroimaging

Antibodies

Antibodies used	Detailed antibody information including vendor, catalog number and clone name was described in the Methods.
Validation	All antibodies were validated by the manufacturer.

Eukaryotic cell lines

Policy information about cell lines

Cell line source(s)	HEK293T cells were purchased from ATCC. All other cells were isolated from transgenic mice or human breast tumors.
Authentication	None of the cell lines used were authenticated.
Mycoplasma contamination	Cells were tested for mycoplasma contamination every 2 months, and only mycoplasma-negative cells were used.
Commonly misidentified lines (See ICLAC register)	No commonly misidentified cell lines were used for this study.

Animals and other organisms

Policy information about studies involving animals; ARRIVE guidelines recommended for reporting animal research

Laboratory animals	Mice were housed under standard specific-pathogen-free (SPF) conditions. ZEB1fl/fl mice were generated in our laboratory. MMTV-PyMT (#002374), MMTV-ErbB2/neu (#005038), MMTV-Wnt1 (#002870), p53fl/fl (#008462) and ROSA-YFP (#006148) transgenic mice were purchased from Jackson Laboratory. FSP1-Cre mice were kindly provided by G. Leone (Ohio State

	University, USA). MMTV-Cre mice (line F, #01XA9) were obtained from US National Cancer Institute (Fredrick, USA). All mice were kept in FVB background with littermates were used in all experiments, and gender-matched littermate controls were used in all experiments.
Wild animals	The study did not involve wild animals.
Field-collected samples	The study did not involve samples collected from the field.
Ethics oversight	All animal experiments were performed in accordance with protocols approved by the Animal Welfare and Ethics Committee of China Pharmaceutical University (AWEC-CPU).

Note that full information on the approval of the study protocol must also be provided in the manuscript.

Flow Cytometry

Plots

Confirm that:

☒ The axis labels state the marker and fluorochrome used (e.g. CD4-FITC).

☒ The axis scales are clearly visible. Include numbers along axes only for bottom left plot of group (a 'group' is an analysis of identical markers).

☒ All plots are contour plots with outliers or pseudocolor plots.

☒ A numerical value for number of cells or percentage (with statistics) is provided.

Methodology

Sample preparation	Primary tumours within the 4th glands were dissected, minced into pieces and digested at 37 °C for 1.5 ~ 2 h in DMEM medium (Thermo Fisher, #11965-092) supplemented with 5% FBS, 10 ng/ml EGF (Peprotech, #315-09), 500 ng/ml hydrocortisone (Sigma-Aldrich, #H0888), 5mg/ml insulin (Sigma-Aldrich, #I9278), 20 ng/ml cholera toxin (Sigma-Aldrich, #C8052), 1% penicillin/streptomycin (Thermo Fisher, #15140122) and collagenase/hyaluronidase (STEMCELL Technologies, #07919). Organoids were collected and incubated with 0.25% trypsin-EDTA (Thermo Fisher, #25200-072) for 1.5 min, 5 mg/ml Dispase (Thermo Fisher, #17105-041) plus 0.1mg/ml DNase (Sigma-Aldrich, #900933) for 5 min, and 0.64% NH4Cl (STEMCELL Technologies, #07850) for 5 min at 37 °C. Following filtration through a 40 µm cell strainer (BD Biosciences, #352340), cells were harvested and resuspended in HBSS buffer containing 0.5% BSA. For flow cytometry analysis, singlecell suspensions were incubated with an antibody cocktail containing CD31 (Biolegend, #102504, 1:100), CD45 (Biolegend, #103104, 1:100) and Ter119 (Biolegend, #116204, 1:100), a secondary biotin-labelled antibody cocktail (STEMCELL Technologies, #19153, 1:100), and magnetic beads (STEMCELL Technologies, #19150, 1:200) for 15 min each on ice. The unbound cells were collected, extensively washed and labelled with FITC-CD49f (BD Biosicences, #561893, 1:200) and APC-CD24 (eBioscience, #11-0242-82, 1:1000) for 30 min, followed by DAPI staining for 5 min before FACS analysis. Luminal, basal and stromal cells were sorted based on the profiles of CD31-CD45-Ter119-(LIN-)DAPI-CD49flowCD24+, LIN-DAPI-CD49f+CD24low and LIN-DAPI-CD49f-CD24-, respectively.
Instrument	Cells were acquired on a BD FACS Verse flow cytometer and sorted on a FACS Aria II flow cytometer.
Software	FlowJo 10 was used to analyse the data.
Cell population abundance	We sorted CD31-CD45-Ter119-DAPI- cells as LIN- subpopulation.
Gating strategy	First standard quadrant gates were set. Subsequently to differentiate DAPI-cells from DAPI+ cells in quadrant 2 gates were set at > 10^4log Fl-2 (CD31-PE-cy7) fluorescence and > 10^4 log Fl-4 (Endomucin-Alexa 647) fluorescence. D31-CD45-Ter119- cells were then sorted according to side scatter and CD31-PE-cy7 fluorescence at > 10^3 log Fl-2 (CD31-PE-cy7) fluorescence after negative selection of leucocyte common antigen CD45 and Ter119 at < 10^3 log Fl-2 (CD45-PE and Ter119-PE) fluorescence.

☒ Tick this box to confirm that a figure exemplifying the gating strategy is provided in the Supplementary Information.
